# CXCL12 Engages Cortical Inhibitory Neurons to Enhance Dendritic Spine Plasticity and Structured Network Activity

**DOI:** 10.1523/JNEUROSCI.2213-24.2025

**Published:** 2025-05-07

**Authors:** Chunta Ho, Jared Luchetta, Bradley Nash, Lindsay K. Festa, James A. Johnson, Ahmet Sacan, Joshua G. Jackson, Antonio Sanz-Clemente, Renato Brandimarti, Olimpia Meucci

**Affiliations:** ^1^Department of Pharmacology and Physiology, Drexel University College of Medicine, Philadelphia, Pennsylvania 19102; ^2^Center for Neuroimmunology and CNS Therapeutics, Institute for Molecular Medicine and Infectious Disease, Drexel University College of Medicine, Philadelphia, Pennsylvania 19102; ^3^Department of Oral Medicine, School of Dental Medicine, University of Pennsylvania, Philadelphia, Pennsylvania 19104; ^4^Department of Neurology, Children’s Hospital of Philadelphia, Philadelphia, Pennsylvania 19104; ^5^School of Biomedical Engineering, Science and Health Systems, Drexel University, Philadelphia, Pennsylvania 19104; ^6^Department of Pharmacy and Biotechnology, University of Bologna, Bologna, 40126, Italy; ^7^Department of Microbiology and Immunology, Drexel University College of Medicine, Philadelphia, Pennsylvania 19102

**Keywords:** chemokine, CXCR4, dendritic spine dynamics, HIV-associated neurocognitive disorder, postsynaptic density

## Abstract

The chemokine CXCL12 is a highly conserved peptide that regulates homeostatic processes in the brain throughout life. Recent work shows that CXCL12 increases dendritic spine density in cortical neurons, which requires activation of CXCL12's receptor CXCR4. This same pathway reverses cortical dendritic spine deficits and cognitive impairment in an animal model of neuroHIV. However, it remained unclear if CXCL12 simply preserved existing spines or also engaged spine plasticity processes that drove network-level adaptations. We therefore tested if CXCL12 could regulate dendritic spine turnover, maturation, clustering, and neuronal network activity in primary rat cortical neurons of either sex using live-cell imaging, confocal microscopy, and multielectrode arrays. Intriguingly, CXCL12-treated neurons formed significantly more new spines than controls, and this outcome was blocked by the CXCR4 antagonist AMD3100. CXCL12 also increased the density of thin spines expressing postsynaptic markers, including postsynaptic density protein 95 (PSD-95), phospho-PSD-95^Ser295^, and GluA1, and allowed neurons to better maintain synaptic PSD-95 puncta size. Thin spines were modestly closer together after CXCL12 treatment, suggesting a possible effect on anatomical spine clustering. These effects translated to structured network activity, as CXCL12 increased spike frequency within network bursts in multielectrode array cultures. Finally, a targeted knockdown of CXCR4 in inhibitory neurons, which mostly lack dendritic spines, prevented CXCL12 from increasing spine density on excitatory neurons. Overall, our findings suggest CXCL12/CXCR4 signaling engages inhibitory neurons along with multiple aspects of spine dynamics and remodeling to shape how broader neuronal networks function.

## Significance Statement

Several neurological disorders accelerate cognitive decline, and there are few effective treatments to slow or reverse cognitive symptoms. Though these disorders often have distinct underlying mechanisms, they typically reduce the density of dendritic spines in brain regions that facilitate learning and memory. We previously reported that the homeostatic chemokine CXCL12 restored dendritic spine density and improved cognitive performance in a rodent model of HIV-associated neurocognitive disorder (HAND), suggesting the pathway holds broadly applicable therapeutic targets. Here, we further uncovered that CXCL12 regulates spine plasticity processes that help spines stabilize and integrate into neuronal networks. These results shed further light on chemokines as intrinsic neuromodulators and their potential to help identify therapeutic targets to restore neuronal function.

## Introduction

CXCL12 is a chemokine that regulates critical processes beyond classical immune cell chemotaxis that are required for life (Mithal et al., 2012). In the CNS, CXCL12 is constitutively expressed and serves as a homeostatic chemokine that regulates developmental and adaptive processes in neurons and glia (Nash and Meucci, 2014). CXCL12 notably guides cortical interneurons along migratory streams during development (Stumm et al., 2003; López-Bendito et al., 2008), but it has more subtle and varied effects in the adult brain. For example, CXCL12 is reported to modulate neurotransmission, regulate neurogenesis, and protect neurons from toxic insults among other actions, which likely interact to promote CNS homeostasis over the lifespan (Li and Ransohoff, 2008; Guyon, 2014). CXCL12 typically achieves these effects by binding and activating the chemokine receptor CXCR4, though it can also be scavenged from the extracellular space via the atypical chemokine receptor ACKR3 (Koch and Engele, 2020). Importantly, these homeostatic signaling pathways are disrupted during CNS diseases like HIV-associated neurocognitive disorder, suggesting they may hold new therapeutic targets to restore neuronal health and function (Irollo et al., 2021; Nickoloff-Bybel et al., 2021; Ellis et al., 2023).

Our work in HIV-1 transgenic rats uncovered severe dendritic spine deficits in layer 2/3 prelimbic cortex neurons that correlated with poor performance in an operant learning task (Festa et al., 2020). Importantly, we could reverse spine and cognitive deficits by treatment with CXCL12 throughout the operant learning procedure, demonstrating that CXCL12's homeostatic and neuroprotective effects extended further than previously known. CXCL12 also increased dendritic spine density in wild-type primary cortical neurons (Pitcher et al., 2014), highlighting the CXCL12/CXCR4 pathway as an endogenous way for neurons to regulate synaptic connectivity. Dendritic spines are highly plastic postsynaptic structures that facilitate learning and memory processes across brain regions (Bourne and Harris, 2007; Heck and Santos, 2023). In response to various stimuli, individual dendritic spines may strengthen or retract/eliminate their connections as needed, and dendrites can also form new spines where none existed before, thereby allowing the local network to adapt via new connections (Zuo et al., 2005; Xu et al., 2009; Yu and Zuo, 2011; Fu et al., 2012). These elegant plasticity processes allow us to learn and remember new information, but they can also be dysregulated and impair cognition in an array of neurocognitive disorders including HAND, Alzheimer's disease, and others (Canet et al., 2018; Nass et al., 2023; McMullan et al., 2024). Therefore, CXCL12's ability to restore dendritic spines might have wider-ranging potential to reverse cognitive impairment.

Recent work, however, suggests that restoring spine density is only part of the puzzle, as cognitive performance is also associated with specific aspects of dendritic spine plasticity, including turnover, clustering, and maturation (Fu et al., 2012; Boda et al., 2014; Bosch et al., 2014; Kastellakis et al., 2015; Runge et al., 2020; Ash et al., 2021; Hedrick et al., 2022). For example, Ccr5^−/−^ mice show enhanced spine turnover in retrosplenial cortex that predicted areas where spine clusters formed as well as contextual learning performance (Frank et al., 2018), suggesting that effective learning requires neurons to rearrange their connections. Furthermore, dendritic spines with postsynaptic density proteins are more likely to resist elimination and integrate with local neuronal networks (Cane et al., 2014), which likely also contributes to learning and memory. Therefore, we suspected that CXCL12/CXCR4 signaling may improve cognition by modulating these aspects of spine plasticity over time. For example, CXCL12/CXCR4 signaling could reverse spine deficits by stabilizing existing spines or helping neurons form new spines over time, and either of these processes could help spines integrate into local networks. Our work demonstrates that CXCL12 does indeed regulate aspects of spine plasticity that improve neuronal network activity and intriguingly utilizes cortical inhibitory neurons to control dendritic spines on local excitatory neurons.

## Materials and Methods

### Cell cultures and treatments

Cortical neurons were isolated from E17 rat embryos from both sexes and mixed to create cultures, as previously described (Brewer et al., 1993; Sengupta et al., 2009). Embryos were obtained from timed-pregnant Holtzman rats (HsdHot:Holtzman SD; Envigo, 003). Neurons were plated onto poly-ʟ-lysine (Sigma-Aldrich P1274) coated 15 mm glass coverslips (3.5 × 10^5^ cells/coverslip) and 60 mm dishes (1 × 10^6^ cells/dish) using Neurobasal medium supplemented with 2% horse serum (Cytiva HyClone, #SH30074.03). After 3 h, the plating medium was replaced with a Neurobasal medium (Thermo Fisher Scientific, #21103049) containing 1% GlutaMAX (Thermo Fisher Scientific, #35050061), 25 μM glutamic acid (Tocris Bioscience, #0218), and 2% B27 (Thermo Fisher Scientific, #17504044). In pure neuronal cultures, 2 μM Ara-C (Sigma-Aldrich, #C6645) was added at DIV2 to reduce glial presence, and >95% of cells were neurons (Festa et al., 2020). Neuron–glial cultures did not receive Ara-C and had 20% glial cells, as determined by GFAP immunostaining. Culture medium was changed on DIV4 and DIV9 with Neurobasal medium containing 1% GlutaMAX and 2% B27, maintaining cultures until DIV21 with weekly half medium changes.

HEK293T cells were cultured with DMEM (Thermo Fisher Scientific, #11995073) with 10% fetal bovine serum (Cytiva HyClone, #SH30070.03HI). Lipofectamine 2000 (Thermo Fisher Scientific, #11668027) was used to transfect HEK293T cells following the manufacturer's instructions.

For CXCL12 (Thermo Fisher Scientific, 400-32A) treatment in the fixed-time point experiment, DIV21 neuronal cultures had their medium replaced with fresh medium (Neurobasal medium containing 1% GlutaMAX and 2% B27) before treatment, and CXCL12 (20 nM) or Vehicle (0.1% BSA/PBS) was directly applied to the cells. After 3 or 6 h, the cells were collected and fixed with 4% PFA, as described in the immunocytochemistry section. For the double CXCL12 (3, 3 h) or Vehicle treatment group, a second treatment of CXCL12 or Vehicle was applied again to the well without replacing the medium.

For the CXCL12 + AMD3100 (MilliporeSigma, #A5602) or AMD3100 alone treatments, cells were pretreated with AMD3100 (100 ng/ml) for 20 min before treating with CXCL12 (20 nM) or Vehicle (0.1% BSA/PBS). All treatments in the live imaging experiment were applied directly to the well. Considering the potential for inefficient diffusion of treatments within the well, all treatment mixtures were adjusted to a final volume of 10 μl, ensuring the desired final concentration when added to the live imaging well containing 1 ml of culture medium. The treatment volume was set to be 10 μl because of its consistent, rapid dispersion speed (less than a minute) in the trypan diffusion test within the well containing 1 ml culture medium.

### Multielectrode array cultures

To prepare multielectrode arrays (MEAs; Multi Channel Systems, #60MEA200/30iR-Ti-gr) for neuronal cultures, the arrays were submerged in 1% tergazyme (Research Products International, #114016) and placed on a rocker at room temperature for 30–60 min. After incubation, any remaining debris was gently removed with a soft-bristled paintbrush, followed by aspiration of the tergazyme. MEAs were then transferred to 100 mm cell culture dishes and washed three times with deionized water for 5 min each. Additional washes were applied as needed to remove residual tergazyme. For short-term storage (1–2 weeks at room temperature), the arrays were placed in new 100 mm dishes filled with sterile distilled water for complete submersion. A day before plating the primary neuronal culture, the arrays were treated with 70% ethanol, and the electrode pads were dried with a lint-free wipe. The arrays were sterilized under germicidal UV light for 1 h and then coated with 1 mg/ml poly-ʟ-lysine in borate buffer and incubated at 37°C overnight. The next day, the MEAs were washed with sterile deionized water and dried before use.

Primary rat cortical neurons were plated on the sterile MEA (1.5 × 10^5^ cells in a 50 μl droplet at the center of the electrode array). Cells were checked for attachment 1.5 h after plating, and then the MEA well was filled with Neurobasal medium containing 2% horse serum. Once cells began to extend processes, the plating medium was aspirated, and the well was filled with Neurobasal medium containing 1% GlutaMAX, 25 μM glutamic acid, and 2% B27. A sterile MEA lid was then added to prevent media evaporation. The following day, the number of attached cells was assessed, and the medium was aspirated and replaced with fresh Neurobasal medium containing GlutaMAX, glutamic acid, and B27. At DIV5, the medium was replaced with Neurobasal medium containing GlutaMAX and B27. Half of the medium was changed every 3–4 d throughout the culture period.

### Multielectrode array recordings

To record neuronal network activity, we used a MEA2100-System with a 60-electrode headstage (Multi Channel Systems, RRID:SCR_014809). MEA temperature was set to 35°C for every recording, while the actual media temperature remained between 32 and 32.5°C. Each MEA underwent a 3 min equilibration period on the headstage before recording. Recordings lasted 6 min, with the total duration outside the incubator not exceeding 10 min. The Multi Channel Experimenter software (Multi Channel Systems, v. 2.17.3.21005) facilitated all raw data collection. Detection threshold for action potential “spikes” was established at −4.5 standard deviations from the baseline noise. The Multi Channel Analyzer software (Multi Channel Systems, v. 2.18.0.21200) was used to analyze the overall activity and synchronous network activity, including network burst number and duration, spike frequency within network bursts, and percent of spikes occurring in bursts.

### Immunocytochemistry

Immunocytochemistry was performed as previously reported (Nash et al., 2019) with modifications. Cortical cultures (DIV21) were first fixed with 2% paraformaldehyde for 10 min, followed by 4% paraformaldehyde for 20 min at 4°C. To stain synaptic proteins within the PSD, we instead used a short fixation (7 min, prewarmed 4% PFA). Cells were then rinsed three times with phosphate-buffered saline (PBS), permeabilized with 0.1–0.5% Triton X-100 (Sigma-Aldrich, #T9284) in PBS for 5–10 min at room temperature depending on the antigen, and then rinsed three times with PBS to wash off the detergent. Cells were then blocked with 5% normal goat serum (Jackson ImmunoResearch, #005-000-121) in PBS containing 0.1% Tween 20 (Sigma-Aldrich, #P9416; PBST) for 30 min at room temperature. For CXCR4 immunocytochemistry, cells were pretreated with lambda protein phosphatase (New England Biolabs, #P7053) before primary antibody incubation. Primary antibodies were applied overnight at 4°C. The next day, cells were washed (three times, 10 min each, PBST) and incubated with secondary antibody in blocking solution for 30 min. We used Alexa Fluor-conjugated phalloidin (Thermo Fisher Scientific, #A1238 and #A12379) in combination with secondary antibodies to visualize dendritic spines. Then, cells were washed (three times, 10 min each, PBST) and counterstained with Hoechst 33342 (Invitrogen, #H3570). Coverslips were mounted with ProLong Gold Antifade Mountant (Thermo Fisher Scientific, #P36930) and cured for 24 h at room temperature before imaging. For immunostaining quantifications, at least 100 cells were counted per area for a total of five areas (*n* ≥ 500 cells) per coverslip and averaged into a single data point. Three coverslips were analyzed per experiment, with three biological replicates/experiments used for the immunostaining quantification.

The following antibodies were used for immunocytochemistry: anti-CXCR4 (Abcam, #ab124824, RRID:AB_10975635), anti-GAD67 (Millipore, #MAB5406, RRID:AB_2278725), anti-GFAP (Millipore, #MAB360, RRID:AB_11212597), anti-GluA1 (Cell Signaling Technology, #13185, RRID:AB_2732897), anti-MAP2 (Abcam, #ab92434, RRID:AB_2138147; Abcam, #ab11267, RRID:AB_297885), anti-pPSD-95 (Ser295, Abcam, #ab76108, RRID:AB_1310621), anti-PSD-95 (Thermo Fisher Scientific, #MA1-046, RRID:AB_2092361), anti-somatostatin-28 (Synaptic Systems, #366006, RRID:AB_2636910), anti-vGluT1 (Synaptic Systems, #135303, RRID:AB_887875), anti-gephyrin (Synaptic Systems, #147011, RRID:AB_887717), and anti-synapsin 1 (Synaptic Systems, #106 103, RRID:AB_11042000).

### AAV transduction of cortical neurons

Cortical neuron transductions were performed as previously reported (Irollo et al., 2023) with the following modifications. For live imaging dendritic spine turnover analysis, neuron–glial cultures (DIV1) were transduced with AAV1-hSyn-EGFP (100 vg/ml). For Cxcr4 gene knockdown in inhibitory neurons, a sequential transduction method was applied. Neuron–glial cultures (DIV1) were first transduced with either AAV9-mDlx-EGFP-miR30a-shCxcr4 (1.5 × 10^5^ vg/ml), AAV9-mDlx-EGFP-miR30a-scramble (1.5 × 10^5^ vg/ml), or AAV9-mDlx-EGFP (1.5 × 10^5^ vg/ml) individually. Then, all groups were transduced with AAV1-hSyn-mScarlet (6 × 10^3^ vg/ml) on DIV14. For all viral transductions on DIV1, the virus-containing medium was replaced with fresh Neurobasal medium with 1% GlutaMAX and 2% B27 on DIV5. The transduced culture was maintained until DIV21. For live imaging of synaptic PSD-95 puncta, cortical neurons were sequentially transduced with AAV1-Syn-PSD-95.FingR-EGFP-CCR5TC (150 vg/ml) and AAV1-hSyn-mScarlet (1.5 × 10^4^ vg/ml) on DIV1 and DIV14, respectively.

### Generation and production of AAV particles

Viral particles of AAV1-Syn-PSD-95.FingR-EGFP-CCR5TC were produced from HEK293T cells via transfection with pAAV-Syn-PSD-95.FingR-EGFP-CCR5TC, AAV2/1 capsid, and pAdDeltaF6 helper virus using the polyethylenimine (PEI) reagents as described in (Challis et al., 2019). Transfected HEK293T cells were collected after 5 d of transfection, and AAV particles were purified using the Takara AAVpro kit (Takara, #6666). Viral particles of AAV9-mDlx-EGFP-mir30a-scramble and AAV9-mDlx-EGFP-mir30a-shCxcr4 were produced by the Penn Vector Core of the University of Pennsylvania.

### Dendritic spines live imaging and data analysis

For the live imaging spine density and turnover experiment, neuron–glial cultures were transduced with AAV1-hSyn-EGFP (100 vg/ml) as described in the AAV transduction section and cultured in glass-bottom 12-well plates (Cellvis, #P12-1.5H-N) until DIV21. A stage-top incubator (Tokai Hit, #INUBTF-WSKM-F1) and its controller (Tokai Hit, #INU-SET-F1) maintained the temperature (36°C) and pH level (95% air:5% CO_2_, 150 ml/min, 0.1 MPa) of the culture during the entire experiment. Dendritic spine images were automatically acquired at designated time points to enhance incubator stability and image quality using an Olympus FluoView FV3000 confocal system (Evident, RRID:SCR_017015) equipped with a 60× objective (PlanApo, NA 1.40). Then, a 2× digital zoom (0.10358 μm/pixel) was applied, and images were acquired with the *Z*-step at 0.15 μm. A built-in function of the FV3000 compensated for *Z*-drift during live imaging at designated time points. To avoid photobleaching and phototoxicity of the imaged dendrite and neuron, a 30 min rest period was placed between imaging sessions. Acquired images were deconvolved with CellSens Dimension Desktop (constrained iterative algorithm; iterations, 3; Olympus, v. 4.2, RRID:SCR_014551), and spines were automatically detected and analyzed by MicroDynamix software (MBF Bioscience, v. 2024.1.1, RRID:SCR_017671), assessing spine density and rates of formation and elimination. The spine turnover rate was determined by dividing the sum of spines formed and eliminated between two time points by the total spine count at each time point (Frank et al., 2018). In each treatment condition, the measured dendrites were at least 100–150 μm in length, and a total 100–120 spines from two consecutive images were analyzed to assess spine turnover. At least one to two dendrites for each treatment condition were analyzed and averaged into a single data point. A total of six biological replicates of each treatment condition were analyzed from individual dissections.

In the live imaging spine stabilization experiment, neuron–glial cultures were transduced with AAV1-Syn-PSD-95.FingR-EGFP-CCR5TC and AAV1-hSyn-mScarlet sequentially in glass-bottom 12-well plates and maintained until DIV21, as described in the AAV transduction section. Image acquisition settings were the same as the spine turnover experiment. The synaptic PSD-95 puncta area on spines was analyzed using the TrackMate (v. 7.11.1) plugin (Tinevez et al., 2017; Ershov et al., 2022) from ImageJ software (v. 1.54f, RRID:SCR_003070). For each condition, we assessed synaptic areas on spines from four neurons. At least six dendritic regions were examined within each neuron, and three to four random spines within each region were analyzed for their synaptic PSD-95 areas. A total of 18–24 spines per neuron were analyzed and then averaged to a single data point for each neuron. Four neurons were analyzed for each condition/experiment, and this procedure was repeated four times across four independent neuronal dissections.

### Dendritic spine density and clustering analysis

Dendritic spine analyses were performed as previously reported with minor modifications (Pitcher et al., 2014; Nash et al., 2019). Neuronal or neuron–glial cultures were fixed on DIV21, and dendritic spines were visualized using Alexa Fluor-conjugated phalloidin or virally expressed fluorescent proteins. Dendritic spine images were acquired using an Olympus FluoView FV3000 confocal microscope equipped with a 100× objective (UPLSAPO 10, NA 1.35) and 2× digital zoom (0.062148 μm/pixel), with a *Z*-step at 0.15 μm. Acquired images were deconvolved with CellSens Dimension Desktop (constrained iterative algorithm; iterations, 3; Olympus, v. 4.2, RRID:SCR_014551), and spines were automatically detected and 3D reconstructed using Neurolucida 360 (MBF Bioscience, v. 2017.01.4, RRID:SCR_016788) software, which facilitated the analysis of spine density and categorization of individual spine types based on established criteria (Rodriguez et al., 2008; Dickstein et al., 2016). For each experimental condition, the dendrite length analyzed was at least 100–150 μm, and at least three to four dendrites on a single coverslip were examined, with a total of three coverslips assessed per condition in a single experiment. The experiment was conducted with a minimum of three independent neuronal dissections (biological replicates) unless otherwise noted.

For dendritic spine clustering analysis, we used MATLAB (MathWorks, v. R2021a, RRID:SCR_001622) to assist with the nearest neighbor index (NNI) analysis and used spine coordinate data from Neurolucida 360. The analysis involved comparing the expected (random) and the average nearest neighbor distance (NND) between consecutive spines on the dendrite. The expected NND was determined using the formula $\mathrm{NNDexpect}=\frac{L}{2N}$ where L represents the dendrite length and N is the number of analyzed spines. The average NND was calculated with $\mathrm{NNDaverage}=\sum_{i=1}^{N}\;\sum_{i\neq j=1}^{N-1}\;\frac{\mathrm{Min}(\mathrm{dij})}{N}$, where Min(dij) denotes the smaller distance for each spine to its consecutive neighboring spines. The NNI was then derived using NNI=NNDaverage/NNDexpect. If the NNI ratio is <1, it suggests that the data set is more clustered than expected in a random distribution. If the ratio is >1, it suggests that the data set is more scattered than random. If the ratio is equal to 1, it suggests that the data set is neither more clustered nor more scattered than expected by chance. The significance of the analyzed NNI was assessed using the standard error calculated as $\mathrm{SE}=\frac{\mathrm{std}(\mathrm{dij})}{\sqrt{N}}$ where std(dij) is the standard deviation of the nearest neighbor distances; and the *t* statistics are calculated as $\frac{\mathrm{NNDaverage}\text{-}\mathrm{NNDexpect}}{\mathrm{SE}}$, which is then converted to a *p*-value for a two-tailed distribution. *p*-values calculated from the NND of multiple dendrites are combined using Fisher's method.

### Combined pre- and postsynaptic staining analysis

Analysis of vGluT1/PSD-95 immunostaining on dendritic spines was performed as described previously (Ma et al., 2008) with minor modifications. Briefly, confocal images of dendrites were acquired and deconvolved as mentioned above. Full synapses were defined as apposed punctate immunofluorescent staining of vGluT1 (presynaptic) and PSD-95 (postsynaptic) in the spine head region. Pre- and postsynaptic staining was quantified with the cell counter plugin from ImageJ software (v. 1.54f, RRID:SCR_003070). The density of dendritic spines containing vGluT1/PSD-95 clusters, or each protein separately, was computed by normalizing spine counts to MAP2^+^ dendrite length.

Analysis of synapsin 1/gephyrin immunostaining on dendrites was performed as previously described (Waataja et al., 2008; Tyagarajan et al., 2011) except that we quantified synapses using the SynapseJ plugin in ImageJ software (Moreno Manrique et al., 2021). SynapseJ performs best when analyzing *z*-stack images, so we acquired confocal images with a 60× objective (PlanApo, NA 1.40, 0.20715 μm/pixel) and a *Z*-step of 0.15 μm, yielding a total thickness of 7–8 μm per image stack. The detection limits for pre- and postsynaptic puncta were optimized to 0.5–2.5 μm to encompass most synapses, and all other settings were kept at their default values. The density of synapsin 1/gephyrin clusters was computed by normalizing cluster counts to the analyzed MAP2^+^ dendrite area. We also checked if the analysis technique could detect increases in synapsin 1/gephyrin puncta as the culture developed from DIV7-21 and found that inhibitory synapse density scaled with time in culture, as expected (data not shown).

In both experiments, four images (synapsin 1/gephyrin) or dendrites (at least 100 μm; vGluT1/PSD-95) were randomly examined from each coverslip. Three coverslips per condition were analyzed in each experiment, yielding 12 images or dendrites per condition per experiment, and the analysis was conducted across three biological replicates.

### Western blot

Western blotting was performed as previously reported (Sengupta et al., 2009). Briefly, cultured neurons from 60 mm dishes were washed with cold PBS and lysed on ice with lysis buffer (50 mM Tris, 1% Triton X-100, 0.5% Na deoxycholate, 0.1% SDS, 150 mM NaCl, 10 mM Na4P2O7, 5 mM EDTA, 1 mM DTT, and protease/phosphatase inhibitors) for 30 min to ensure complete lysis. Lysates were then centrifuged at 20,000 × *g* for 15 min at 4°C, and the supernatant was collected. Lysate protein concentration was determined using the BCA protein assay (Thermo Fisher Scientific, #23225). For SDS-PAGE, an equal amount of protein (30–40 μg) was loaded into each lane and subsequently transferred to a PVDF membrane (Millipore, #IPVH00010) for immunoblotting.

The following antibodies were used for immunoblotting: anti-β-actin (1:3,000, Sigma-Aldrich, #A5316, RRID:AB_476743), anti-CXCR4 (1:100, Abcam, #ab124824, RRID:AB_10975635), anti-PSD-95 (1:1,000, Thermo Fisher Scientific, #MA1-046, RRID:AB_2092361), and anti-GFP (1:5,000, Aves Labs, #GFP-1010, RRID:AB_2307313).

### Nucleofection of cortical neurons

Nucleofection was performed as previously reported (Sengupta et al., 2009). Briefly, 2 × 10^6^ cells from cortical neuron dissections on DIV1 were utilized for each nucleofection experiment (Lonza, #VPG-1003) using the Lonza Nucleofector II device (Lonza, RRID:SCR_022262). Between 1 and 2 μg of plasmid DNA of pAAV-mDlx-CXCR4 was introduced into the nucleofection mixture, and the device's preset protocol G-013 was used for electroporation. Immediately after, Neurobasal medium supplemented with 2% horse serum was added to the cuvette, which was then incubated for 5–10 min to enhance cell recovery. Subsequently, cells were cultured on coverslips for 2–3 h before the medium was replaced with fresh Neurobasal medium enriched with 1% GlutaMAX, 25 μM glutamic acid, and 2% B27 as described in the primary neuronal culture section and maintained as the neuron–glial culture condition until DIV21 for immunocytochemistry experiments. The nucleofection efficiency, indicated by kit-provided pmaxGFP (Lonza, #VPG-1003) expression, was between 50–60% postnucleofection in neuron–glial cultures.

### In vivo CXCL12 treatment and dendritic spine analysis

Adult F344/NHsd male rats (Envigo Laboratories, 010) were used for ICV injections and dendritic spine analysis. The rats were housed individually in isolation within our Association for Assessment and Accreditation of Laboratory Animal Care–accredited barrier facilities, following National Institutes of Health guidelines and with approval from the Institutional Animal Care and Use Committee.

Intracerebroventricular injections (ICV) were performed as previously reported (Festa et al., 2020). Adult male rats (F344/NHsd, 4–5 months old) were under anesthesia with isoflurane and ketamine/xylazine administration (50 and 10 mg/kg, i.p., respectively) and underwent stereotaxic implantation of cannulas (AP, 0.96 mm; ML, 2.00 mm; DV, 3.5 mm). Following surgery, the animals were allowed a 7 d recovery period and then received daily infusions of either Vehicle solution (0.1% BSA/PBS) or CXCL12 (5 ng/μl in 0.1% BSA/PBS, total volume 25 ng/5 μl/dose) for up to 3 d before the dendritic spine analysis.

Dendritic spines in fixed rat brain tissue were analyzed as previously described in Festa et al. (2020) and originally described in Seabold et al. (2010). Briefly, dendrites at least 100–150 μm from layer 2/3 pyramidal neurons in the prelimbic cortex were imaged using the Zeiss LSM 5 confocal system equipped with a 63× objective (PlanApo 63×/1.4 oil, 0.076 μm/pixel) with a *Z*-step at 0.1 μm. Spines were then 3D reconstructed and analyzed as described above using Neurolucida 360 software. Eight dendrites from eight individual neurons were analyzed and averaged to yield one data point per rat, with a total of four rats analyzed per treatment group.

### Materials

pAAV-hSyn-EGFP (Addgene viral prep #50465-AAV; RRID:Addgene_50465) was a gift from Bryan Roth, pAAV-Syn-PSD-95.FingR-EGFP-CCR5TC (Addgene plasmid #125693; RRID:Addgene_125693) was a gift from Xue Han (Bensussen et al., 2020), pAAV-hSyn-mScarlet (Addgene viral prep #131001-AAV1; RRID:Addgene_131001) was a gift from Karl Deisseroth (Marshel et al., 2019), pAAV-mDlx-GFP-Fishell-1 (Addgene viral prep #83900-AAV9; RRID:Addgene_83900) was a gift from Gordon Fishell (Dimidschstein et al., 2016), pAAV2/1 (Addgene plasmid #112862; RRID:Addgene_112862) and pAdDeltaF6 (Addgene plasmid #112867; RRID:Addgene_112867) were gifts from James M. Wilson.

To generate rCXCR4-EGFP and rCXCR4 ΔCT-EGFP plasmids, sequences of full-length and C-terminally truncated CXCR4 were amplified from plasmid RG80342-ACG (Sino Biological) and cloned into pUltraHot (Addgene plasmid #24130, a gift from Malcolm Moore; RRID:Addgene_24130). AAV-mDlx-CXCR4 was constructed by introducing the CXCR4 sequence, amplified from the rCXCR4-EGFP plasmid, into pAAV-mDlx-EGFP-Fishell-1.

To generate AAV-mDlx-EGFP-mir30a-shRNA constructs, we cloned a shRNA targeting the rat Cxcr4 gene (forward: 5′-cccacttaccaaagacatatatccgagatatattctttgcgtaagtgtttttg-3′; reverse: 5′ aattcaaaaacacttaccaaagacatatattgagatatattctttgcgtaagtg-3′) into a custom LV-Cre-ON-EGFP-miR30a-scramble vector (Vigene Biosciences), replacing its original scramble sequence. The miR30a-scramble and miR30a-shCxcr4 sequences were then amplified and inserted into the pAAV-mDlx-EGFP-Fishell-1. All plasmids constructed for this study were generated using the NEBuilder HiFi DNA Assembly Cloning Kit (New England Biolabs, E5520S), with oligonucleotides synthesized by IDT DNA. DNA amplification was conducted with the high-fidelity Q5 polymerase (New England Biolabs, M0492S).

### Experimental design and statistical analysis

In vitro experiments were conducted using neurons from distinct litters of E17 r­­at embryos and presented as mean ± SEM in bar graphs and median ± SEM in violin plots without outlier removal. Dendritic spine density analysis from rats receiving daily ICV injections was presented as mean ± SEM in bar graphs. Two-way ANOVA with mixed model method and Tukey's post hoc was used to analyze dendritic spine density changes over time in live imaging experiments. Two-way ANOVA with Tukey's post hoc was used to analyze synaptic PSD-95 area/spine changes, dendritic spine density changes (PSD-95, pPSD-95, and GluA1) and Cxcr4 knockdown cortical cultures, spike frequency in network burst/MEA and burst duration/MEA between the experimental and control groups, and CXCR4^+^ cell percentage in inhibitory and excitatory neuron populations.

One-way ANOVA with Tukey's post hoc was used to analyze dendritic spine metrics (density changes, formation, elimination, and turnover) in live imaging experiments at specific time points and Dunnett's test for dendritic spine density changes with ICV injection. One-way ANOVA with Tukey's post hoc was used to analyze synapsin 1/gephyrin staining, MAP2^+^ dendrite area, the percentage of CXCR4^+^ neurons in excitatory or inhibitory neuronal populations, and CXCR4 immunostaining intensity and fold change. Repeated measures one-way ANOVA with Tukey's post hoc was used to analyze activity changes in MEA optimization experiments, including overall activity, network burst, spike frequency in network bursts, percentage of spines in network bursts, and network burst duration. For varied data distribution, the Kruskal–Wallis test with Dunn's correction was used to analyze endogenous PSD-95 puncta size (untransduced, EGFP, and PSD-95.FingR-EGFP)

An unpaired *t* test was used to analyze the difference in NNI, MAP2^+^ dendrite length or area, vGluT1^+^/PSD-95^+^ immunostaining on overall spines or PSD-95^+^ spines between experimental and control groups, and the percentage of CXCR4^+^ neurons in neuron–glial and neuronal cultures. The Mann–Whitney *U* test was used to analyze spine density changes in fixed-time point experiments, including single (3, 6 h) and double (3, 3 h) CXCL12 treatment, and the Kolmogorov–Smirnov test was used to analyze the spine head diameter distribution between the experimental and control groups. The Mann–Whitney *U* and Kolmogorov–Smirnov tests were chosen because they are suitable for the varied data distribution in the CXCL12-treated group. Statistical significance was set at *p* < 0.05, with all analyses performed using GraphPad Prism (v. 10.4, RRID:SCR_002798). Figures show all the comparisons that reached statistical significance, and *p*-values are designated as *p ≤ 0.05, **p ≤ 0.01, ***p ≤ 0.001, and ****p ≤ 0.0001.

**Figure 1.**
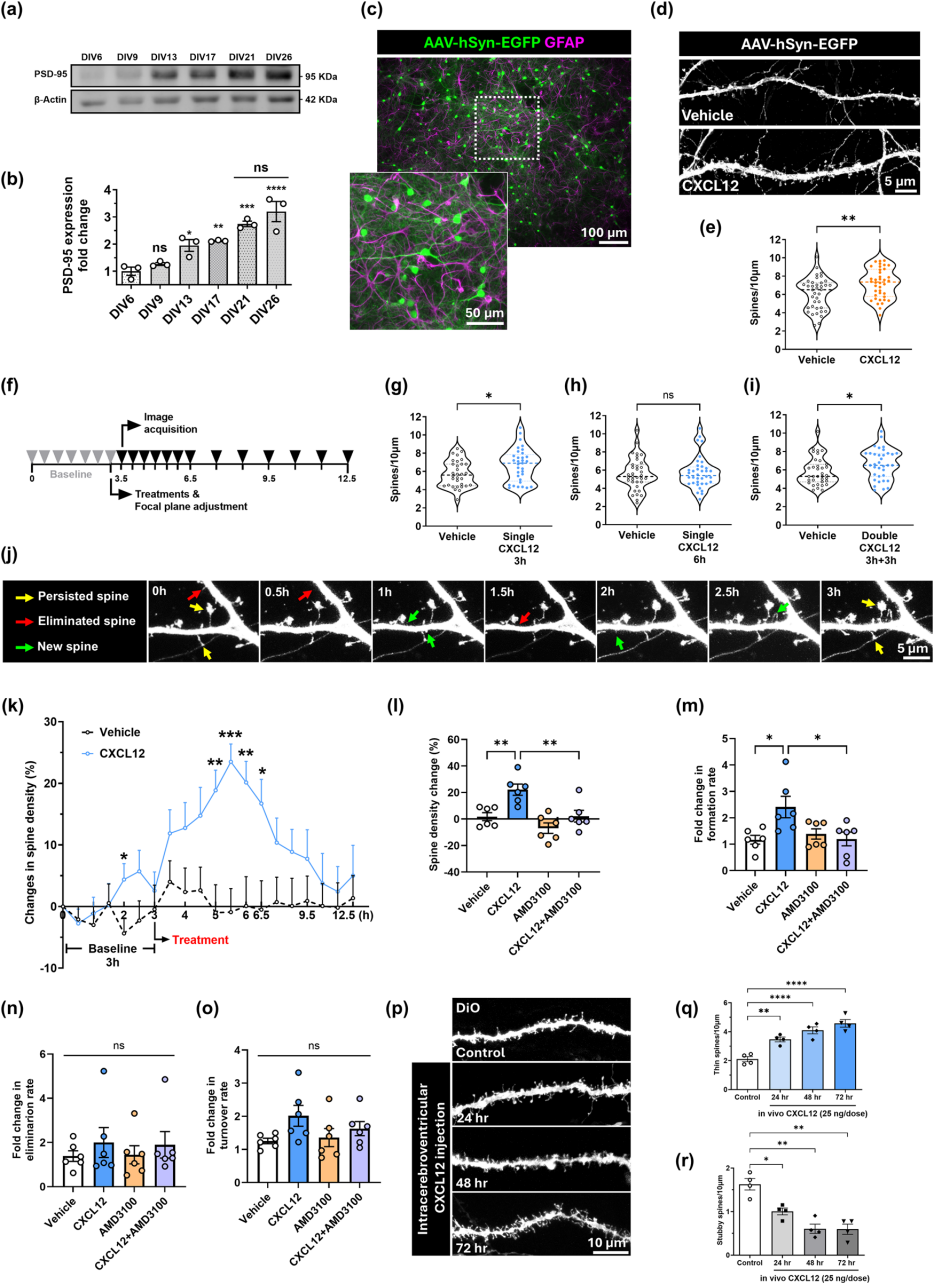
CXCL12/CXCR4 signaling promotes spine formation. ***a***, Representative Western blot of PSD-95 expression from lysates of neuronal cultures over time (DIV6, 9, 13, 17, 21, and 26). ***b***, Quantification of PSD-95 expression fold change from Western blots. ***c***, Representative fluorescence image of DIV21 cortical neuron–glial cultures transduced with AAV1-hSyn-EGFP (green) and immunostained for GFAP (magenta). The inset shows an area of higher magnification. ***d***, Dendritic spine morphology in AAV-transduced cortical neurons treated with Vehicle (0.1% BSA/PBS) or CXCL12 (20 nM). ***e***, Quantification of dendritic spine density in DIV21 AAV-transduced cortical cultures treated with Vehicle or CXCL12. ***f***, The experimental timeline for spine live imaging. Gray arrowheads mark the baseline phase, and black arrowheads mark imaging sessions after treatments. ***g***, Dendritic spine density in cortical neurons treated with a single dose of Vehicle or CXCL12 after 3 h, (***h***) after 6 h, and (***i***) after two CXCL12 treatments at 0 and 3 h. ***j***, Representative images acquired during baseline live imaging. Arrows show spine formation (green), elimination (red), and persistence (yellow). ***k***, Quantified spine density changes during live imaging of neuronal cultures treated with Vehicle or CXCL12. ***l***, Spine density changes following CXCL12 treatment, with or without pretreatment with the CXCR4 antagonist AMD3100 (100 ng/ml). ***m***, CXCL12 and AMD3100 effects on spine formation rate, (***n***) spine elimination rate, and (***o***) spine turnover rate after 3 h. ***p***, Diolistically (DiO) stained dendrites from prelimbic cortex of 4–5-month-old male F344 rats that received daily intracerebroventricular infusions of Vehicle (0.1% BSA/PBS, 5 μl) or CXCL12 (25 ng/5 μl) for up to 3 d. ***q***, Density of thin spines and (***r***) stubby spines from Vehicle or CXCL12-treated F344 rats.

**Figure 2.**
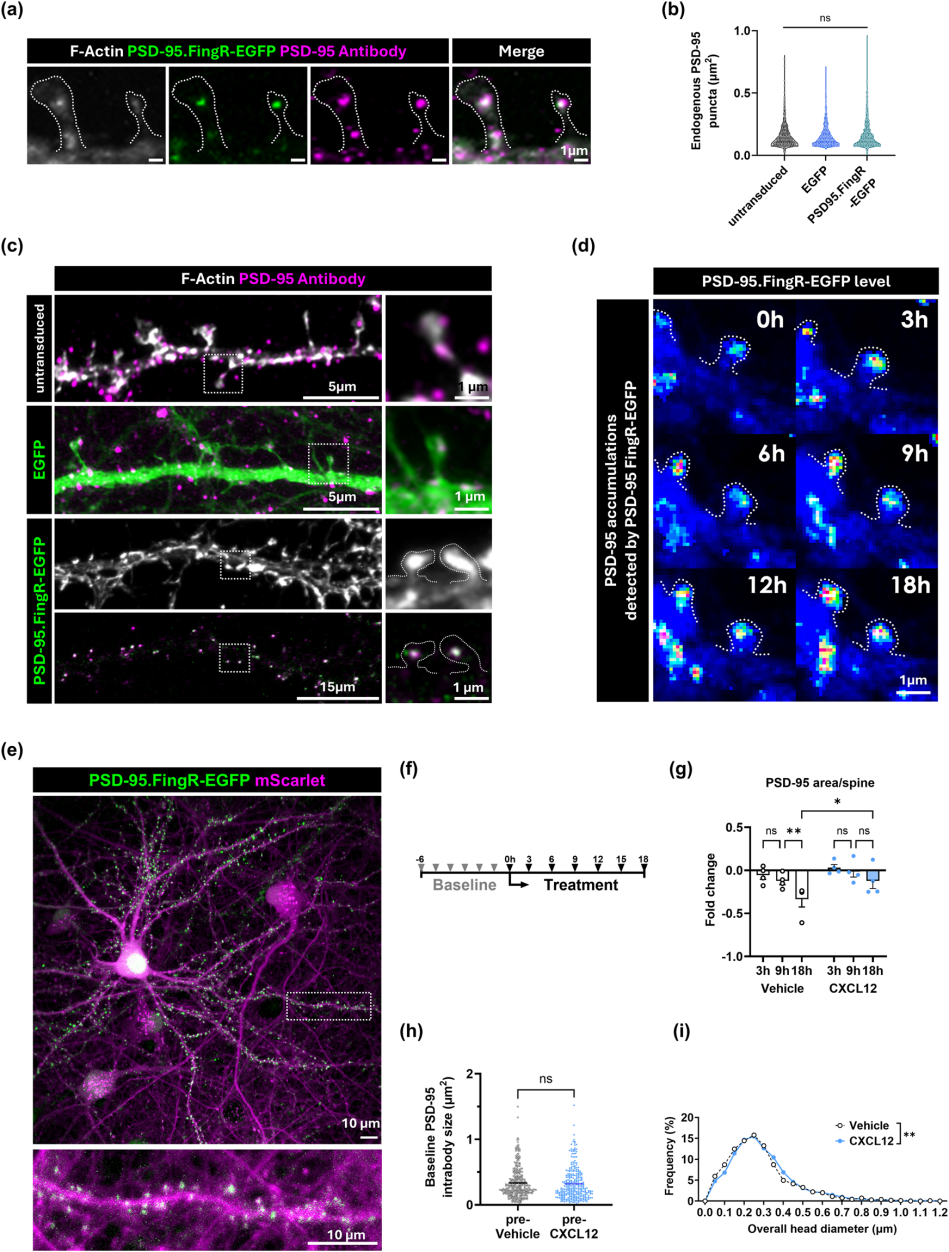
CXCL12 stabilizes dendritic spines. ***a***, Representative fluorescence image of a DIV21 cortical culture expressing a transduced PSD-95 intrabody (green, PSD-95.FingR-EGFP) and immunostained with a PSD-95 antibody (magenta) within dendritic spines (gray, F-actin). ***b***, Quantification of antibody-labeled PSD-95 puncta area in untransduced, EGFP-transduced, and PSD-95 intrabody groups. ***c***, Representative immunofluorescence images of dendrites and spines expressing PSD-95 intrabody (green) or EGFP (green) in DIV21 neuron–glial cultures costained with PSD-95 antibody (magenta) or F-actin (gray). ***d***, Heatmap intensity of PSD-95 intrabody level in a DIV21 neuron–glial culture. ***e***, Representative live imaging frame of a cortical neuron dually transduced with AAV1-Syn-PSD-95.FingR-EGFP and AAV1-hSyn-mScarlet in DIV21 neuron–glial cultures. ***f***, Timeline for live imaging of endogenous synaptic PSD-95 using the PSD-95 intrabody. Arrowheads indicate imaging sessions, conducted every hour during the baseline period and every 3 h during the treatment observation. ***g***, Quantification of the PSD-95 intrabody puncta area in Vehicle and CXCL12-treated groups. ***h***, Baseline comparison of the PSD-95 intrabody puncta area between pre-Vehicle and pre-CXCL12 groups. ***i***, Quantification of overall spine head diameter in DIV21 neuronal cultures treated with Vehicle or CXCL12.

**Figure 3.**
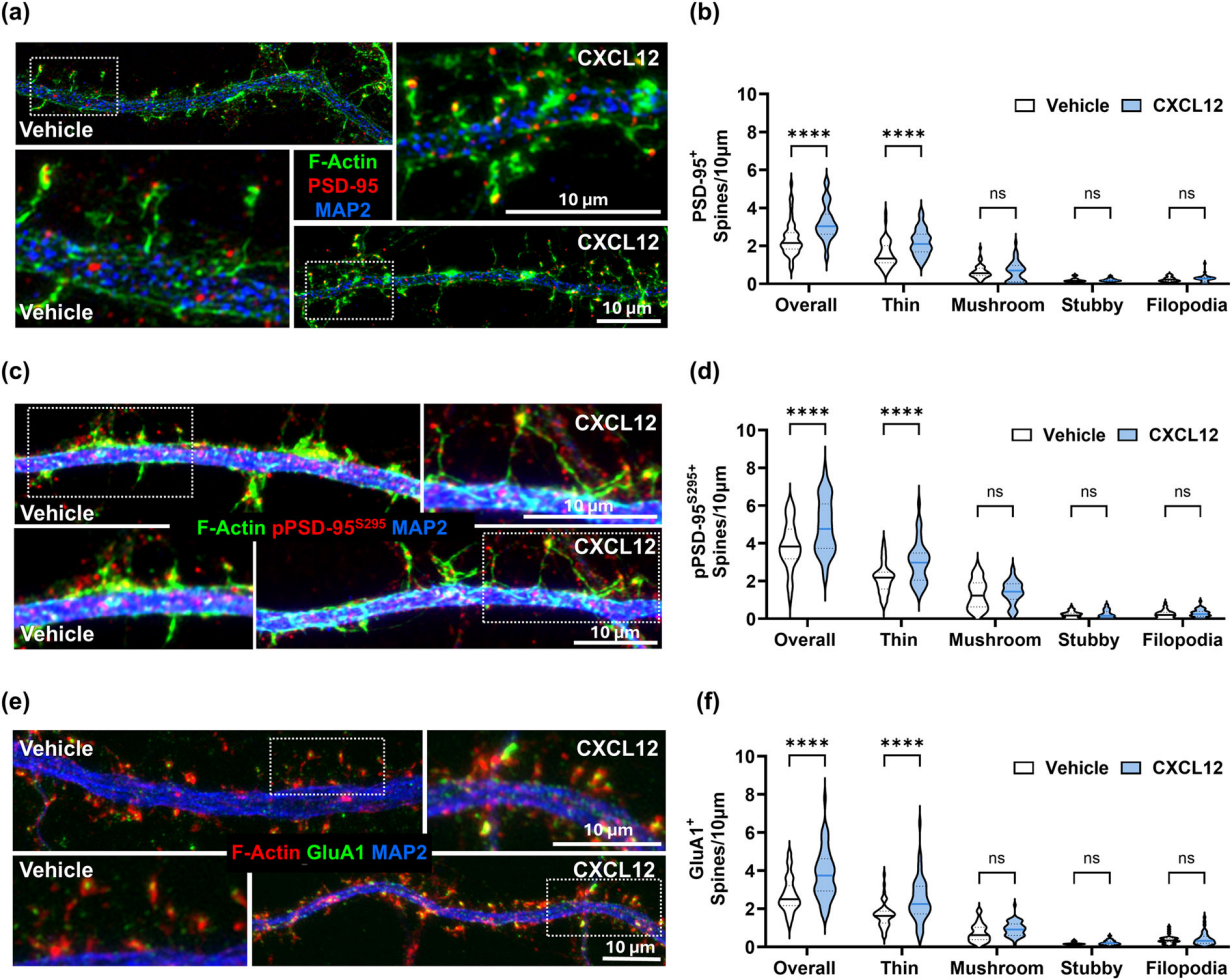
CXCL12 increases postsynaptic proteins in spines. Representative images of DIV21 neuronal cultures treated with CXCL12 or Vehicle for 3 h and fixed/stained for MAP2 (blue), F-actin (phalloidin, green), and either (***a***) PSD-95 (red), (***c***) pPSD-95^Ser295^ (red) or (***e***) GluA1 (green, phalloidin in red). Quantification of how CXCL12 treatment altered the densities of spines positive for (***b***) PSD-95, (***d***) pPSD-95^Ser295^, and (***f***) GluA1 from the overall spine population and separated by spine type (thin, mushroom, and stubby) or filopodia.

**Figure 4.**
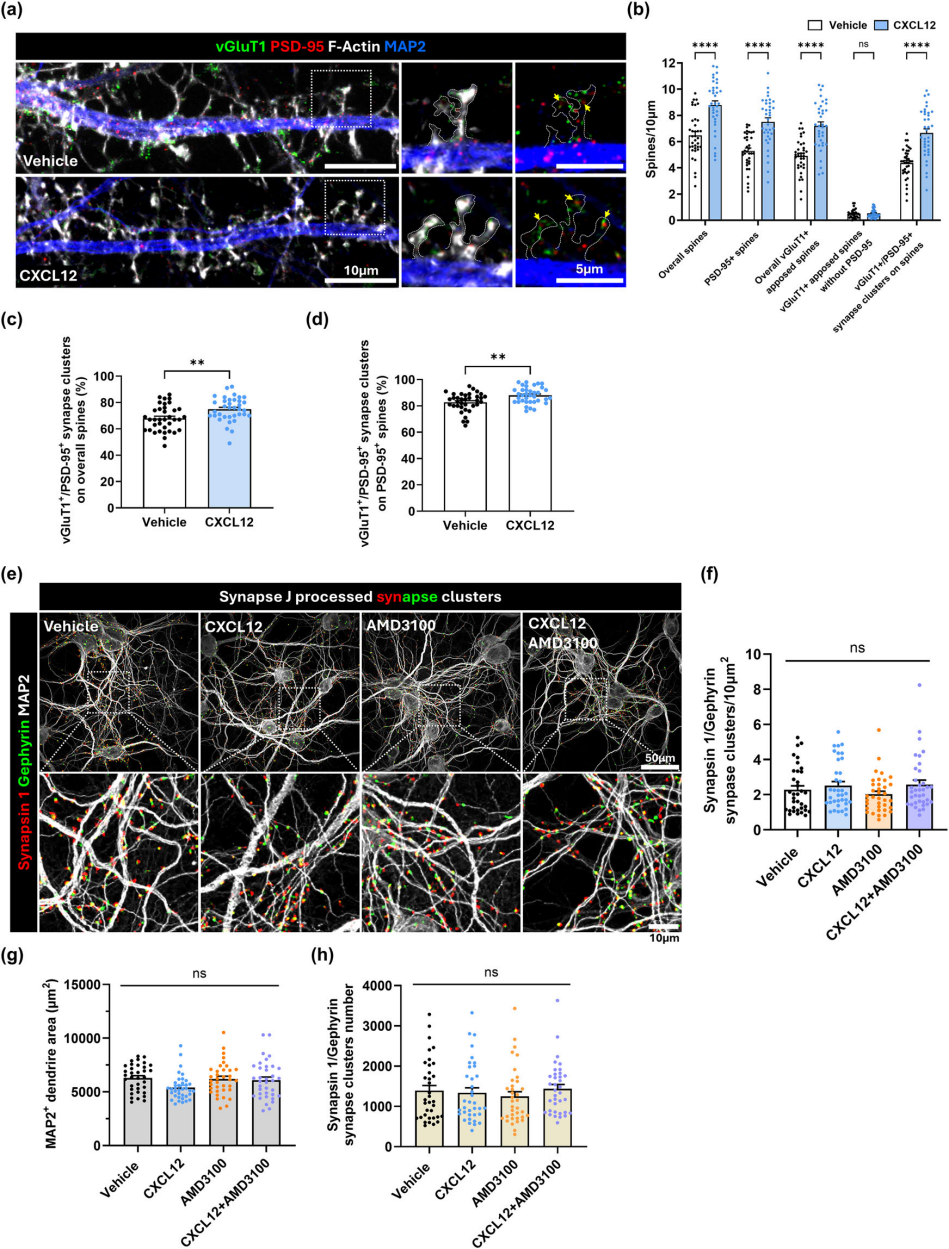
CXCL12 effects on excitatory and inhibitory synaptic proteins. ***a***, Representative images of DIV21 neuronal cultures treated with CXCL12 or Vehicle for 3 h and fixed/stained for MAP2 (blue), F-actin (phalloidin, white), PSD-95 (red), and vGluT1 (green). ***b***, Quantifications from CXCL12 and Vehicle groups of overall dendritic spine density and those spines containing PSD-95, vGluT1, or clusters of both proteins. vGluT1-only apposed spines are immature spine types that do not contain PSD-95 but are apposed to vGluT1 staining. ***c***, Quantification of the percentage of overall spines with vGluT1/PSD-95 staining clusters. ***d***, Quantification of the percentage of PSD-95 containing spines with vGluT1/PSD-95 staining clusters. ***e***, Representative images of DIV21 neuronal cultures treated with Vehicle, CXCL12, AMD3100, or the combination for 3 h and fixed/stained for MAP2 (white), synapsin 1 (red), and gephyrin (green). ***f***, Quantifications of the density of synapsin 1/gephyrin staining clusters along MAP2^+^ dendrites. ***g***, Quantification of the average area of MAP2^+^ dendrites measured for each treatment group. ***h***, Quantification of the average numbers of synapsin 1/gephyrin clusters counted from each treatment group.

**Figure 5.**
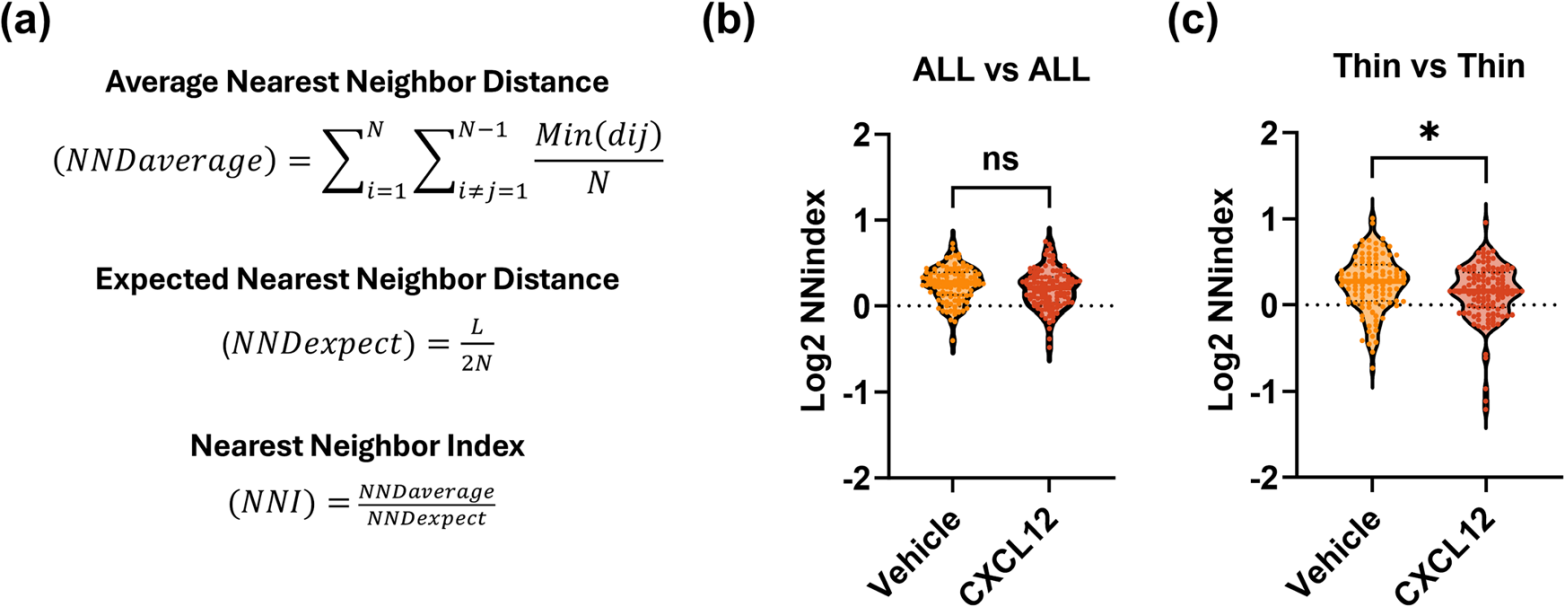
CXCL12 subtly promotes spine clustering. ***a***, The nearest neighbor index formula. ***b***, ***c***, Comparison of NNI in (***b***) overall spine populations and (***c***) thin spine populations from Vehicle and CXCL12-treated DIV21 neuronal cultures.

**Figure 6.**
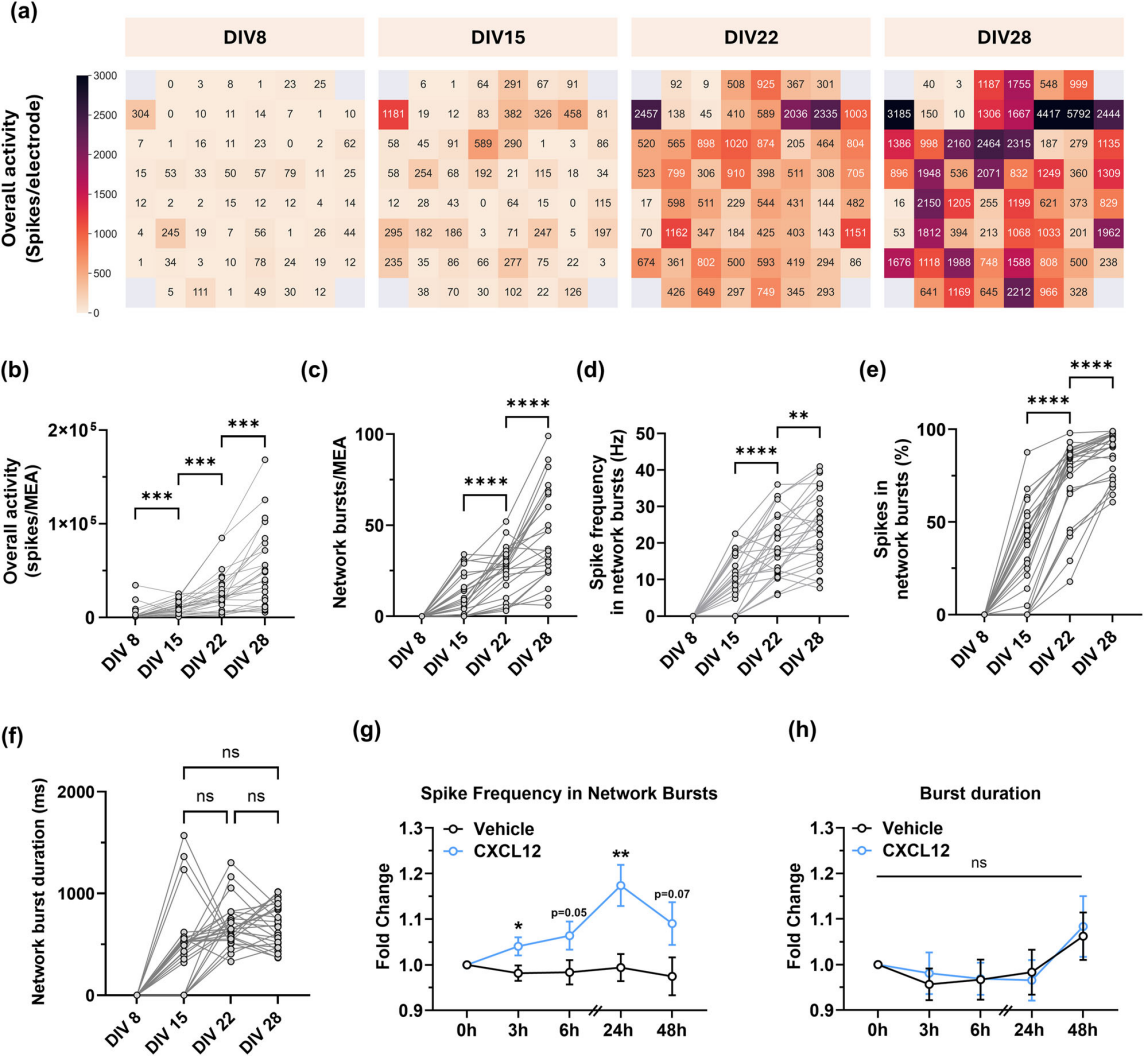
CXCL12 enhances structured network activity by increasing spike frequency in network bursts. ***a***, Heatmaps showing the number of spikes recorded at each electrode in a representative MEA cortical neuron–glial culture over time. ***b***–***f***, Quantifications of key network parameters over the same time span, including (***b***) overall activity, (***c***) network bursts, (***d***) spike frequency within network bursts, (***e***) percentage of spikes in network bursts, and (***f***) network burst duration, illustrating a consistent and significant weekly development in MEA neuron–glial cultures up to 28 d in vitro. ***g***, Quantification of CXCL12 effects on spike frequency within bursts in DIV28 MEA cultures. ***h***, Quantification of the burst duration between the Vehicle and CXCL12-treated DIV28 MEA cultures over the same time points.

**Figure 7.**
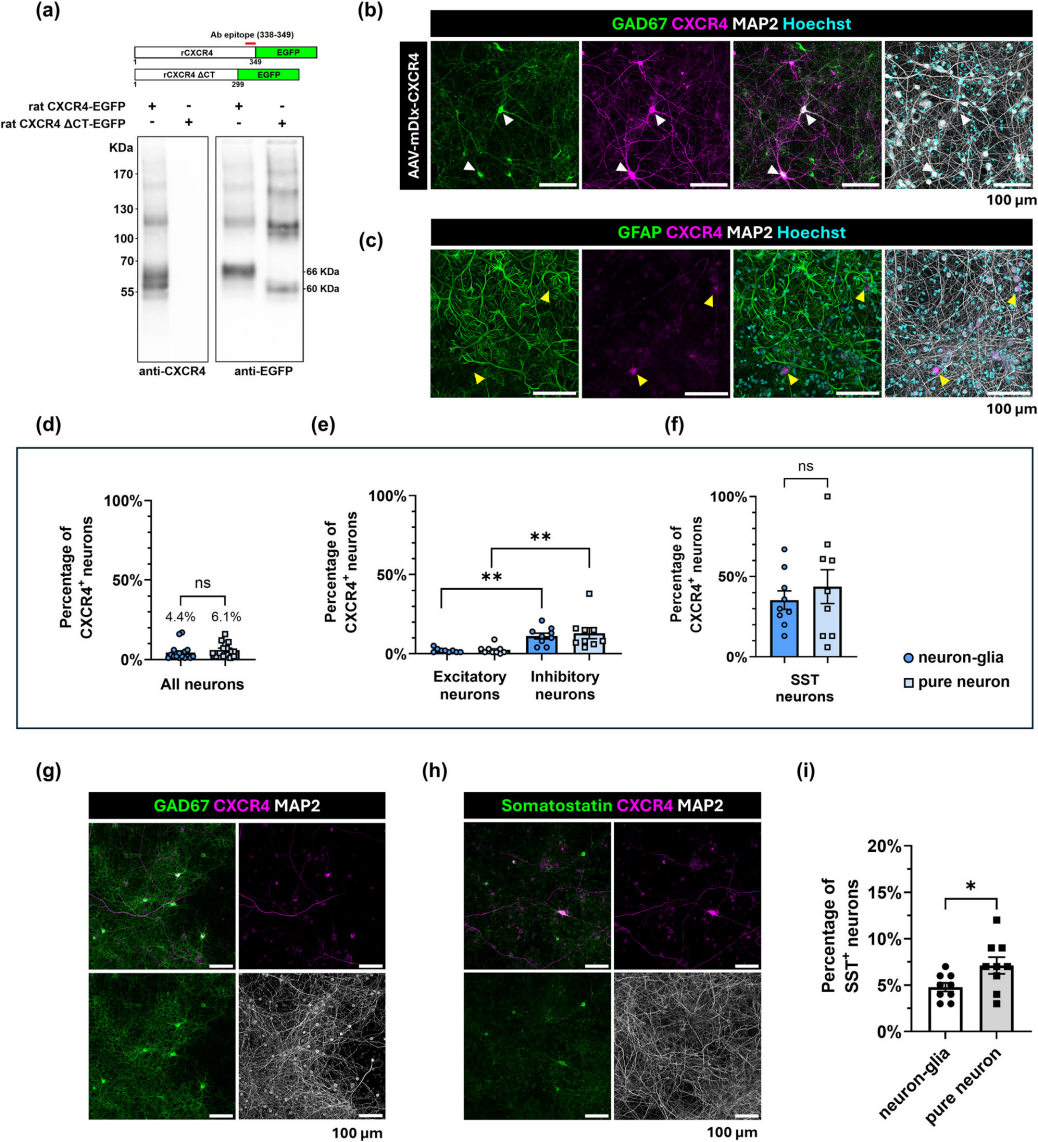
CXCR4 is enriched in inhibitory neurons of cortical neuron–glial cultures. ***a***, Specificity of the CXCR4 antibody in Western blot of HEK293T cells expressing rat CXCR4-GFP, with and without the antibody binding epitope. ***b***, Specificity of the CXCR4 antibody in immunocytochemistry of neuron–glial cortical cultures nucleofected with the AAV-mDlx-CXCR4 plasmid. CXCR4 (magenta)-stained GAD67-positive inhibitory neurons (green), overlapping with the neuronal marker MAP2 and Hoechst 33342 (gray and cyan). ***c***, Neuron–glial cortical cultures immunostained for CXCR4 (magenta), the astrocyte marker GFAP (green), the neuronal marker MAP2 (gray), and Hoechst 33342 (cyan). ***d***, Quantification of the percentage of CXCR4-positive neurons in the total neuronal population, (***e***) excitatory neurons (GAD67-negative) and inhibitory neurons (GAD67-positive), and (***f***) SST-positive neurons in pure neuronal and neuron–glial cultures. ***g***, Representative image of GAD67 (green) and CXCR4 (magenta) distribution overlapping with the neuronal marker MAP2 (gray). ***h***, Representative image of CXCR4 (magenta) staining overlapping with SST-positive neurons (green) and the neuronal marker MAP2 (gray). ***i***, Quantification of the percentage of SST-positive neurons within the total neuronal population from neuronal and neuron–glial cultures.

**Figure 8.**
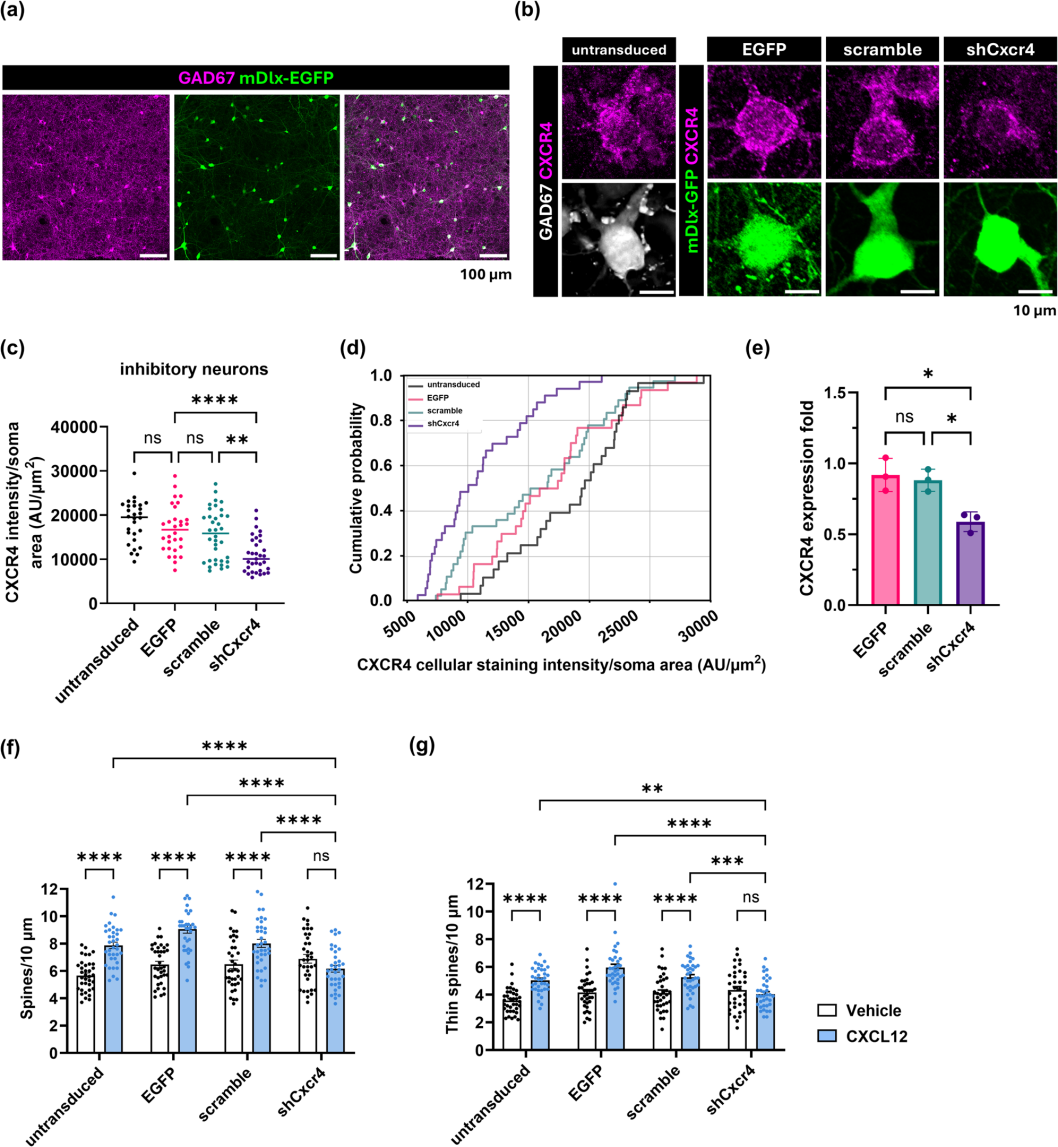
CXCR4 knockdown in inhibitory neurons blocks CXCL12 from regulating dendritic spines. ***a***, Representative images from a neuron–glial culture transduced with AAV-mDlx-EGFP and immunostained for GAD67 (magenta), demonstrating the viral construct targets GAD67^+^ inhibitory neurons. ***b***, Representative CXCR4 protein expression (magenta) in inhibitory neurons from untransduced cultures (GAD67, white) and cultures transduced with AAV-mDlx-shCxcr4 or control constructs (EGFP, green). ***c***, Quantification of CXCR4 immunofluorescence intensity from individual inhibitory neurons of AAV-mDlx-shCxcr4–transduced, control-transduced, and untransduced neuronal cultures. CXCR4 staining was quantified in GAD67^+^ somata of untransduced cultures and GFP^+^ somata of transduced cultures. ***d***, Empirical cumulative distribution function of CXCR4 staining intensity from individual inhibitory neurons of each experimental group. ***e***, Normalization to the untransduced group indicated that approximately half of the CXCR4 expression was knocked down in the shCxcr4-transduced inhibitory neurons. ***f***, CXCL12 failed to increase spine density in AAV-mDlx-shCxcr4–transduced cultures, compared with scramble and EGFP controls as well as untransduced cultures. ***g***, CXCL12 also failed to increase thin spine density in AAV-mDlx-shCxcr4–transduced cultures compared with controls.

*N* (biological replicates: neuronal dissections) and *n* (technical replicates: analyzed samples) values of each quantification were detailed as follows: Figure 1*b* *N* = 3; Figure 1*e* *N* = 3, *n* = 39 dendrites for Vehicle, *n* = 40 dendrites for CXCL12; Figure 1*g* *N* = 3, *n* = 32 dendrites for Vehicle, *n* = 33 dendrites for Single CXCL12 3 h; Figure 1*h* *N* = 3, *n* = 35 dendrites for Vehicle, *n* = 36 dendrites for Single CXCL12 6 h; Figure 1*i* *N* = 3, *n* = 35 dendrites for Vehicle, *n* = 35 dendrites for Double CXCL12 3 + 3 h; Figure 1*k* *N* = 4, *n* = 11 dendrites for Vehicle, *n* = 8 dendrites for CXCL12; Figure 1*l–n* *N* = 6, *n* = 10 dendrites for Vehicle, *n* = 9 dendrites for CXCL12, *n* = 7 dendrites for AMD3100, *n* = 11 dendrites for CXCL12 + AMD3100; Figure 1*p,q* *N* = 4 animals and 32 dendrites for each control, 24, 48 and 72 h treatments; Figure 2*b* *N* = 3, *n* = 909 puncta for untransduced, *n* = 1,195 for EGFP, *n* = 1,252 for PSD-95.FingR-EGFP; Figure 2*g* *N* = 4, *n* = 16 neurons for Vehicle, *n* = 15 neurons for CXCL12; Figure 2*h* *N* = 3, *n* = 317 puncta for pre-Vehicle, *n* = 300 puncta for pre-CXCL12; Figure 1*i* *N* = 8, *n* = 2,083 spines for Vehicle, *n* = 3,286 spines for CXCL12; Figure 3*b* *N* = 4, *n* = 46 dendrites for Vehicle, *n* = 46 dendrites for CXCL12; Figure 3*d* *N* = 3, *n* = 37 dendrites for Vehicle, *n* = 39 dendrites for CXCL12; Figure 3*f* *N* = 3, *n* = 47 dendrites for Vehicle, *n* = 52 dendrites for CXCL12; Figure 4*b* *N* = 3, *n* = 36 dendrites for both Vehicle or CXCL12 in the overall spines, PSD-95^+^ spines, overall vGluT1^+^- apposed spines, and vGluT1^+^/PSD-95^+^ synapse clusters on spines; *n* = 30 dendrites for Vehicle and *n* = 32 dendrites for CXCL12 in the vGluT1^+^-only apposed spines (PSD-95^−^); Figure 4*c–e* *N* = 3, *n* = 36 dendrites for both Vehicle or CXCL12; Figure 4*g–i* *N* = 3, *n* = 35 images for Vehicle, *n* = 36 images for CXCL12, AMD3100, or CXCL12 + AMD3100; Figure 5*b* *N* = 7, *n* = 110 dendrites for Vehicle, *n* = 119 dendrites for CXCL12; Figure 6*b–f* *N* = 4, *n* = 26 MEA cultures for each condition, i.e., DIV8, DIV15, DIV22, and DIV28; Figure 6*g,h* *N* = 4, *n* = 26 MEA cultures for Vehicle, *n* = 27 MEA cultures for CXCL12; Figure 7*d,g* *N* = 3, counted MAP2^+^ cells, *n* = 6,267 for neuron–glial cultures, *n* = 3,376 for neuronal cultures; Figure 7*f* *N* = 3, counted GAD67^+^ cells, *n* = 617 for neuron–glial cultures, *n* = 466 cells for neuronal cultures; Figure 7*i* *N* = 3, counted somatostatin^+^ cells, *n* = 251 for neuron–glial cultures, *n* = 163 for neuronal cultures; Figure 8*c,e* *N* = 3, CXCR4^+^ cells, *n* = 28 cells for untransduced, *n* = 30 cells for EGFP, *n* = 36 cells for scramble, *n* = 33 cells for shCxcr4; Figure 8*f,g* *N* = 3, *n* = 36 dendrites for both Vehicle or CXCL12 in the untransduced, scramble; *n* = 36 dendrites for Vehicle, *n* = 35 for CXCL12 in the EGFP; *n* = 36 dendrites for Vehicle, *n* = 35 dendrites for CXCL12 in the shCxcr4.

## Results

### CXCL12/CXCR4 signaling regulates dendritic spine formation

Our previous work showed that CXCL12/CXCR4 signaling increases dendritic spine density in cortical neurons both in vitro and in vivo (Pitcher et al., 2014; Festa et al., 2020). However, it was unclear if this was due to more stabilized spines or enhanced spine turnover, an important mechanism of spine plasticity that promotes learning and memory. We wanted to examine these possibilities in primary rat cortical neurons with established neuronal networks, so we first tracked the synaptic marker PSD-95 over the culture lifespan. PSD-95 expression increased from DIV13 to DIV26, as expected from a developing culture (Fig. 1*a,b*; DIV6: 1.0-fold ± 0.15; DIV9: 1.27-fold ± 0.05, *p* = 0.7857; DIV13: 1.95-fold ± 0.22, *p* = 0.0394; DIV17: 2.1-fold ± 0.02, *p* = 0.0138; DIV21: 2.7-fold ± 0.1, *p* = 0.0004; DIV26: 3.2-fold ± 0.4; pairwise comparisons to DIV6; *F*_(5,12) _= 19.22, interaction *p* < 0.0001, ANOVA). PSD-95 expression reached a plateau between DIV21 and DIV26, suggesting the cultures established a network phenotype at this point (Fig. 1*b*; DIV 21: 2.7-fold ± 0.17 vs DIV26: 3.2-fold ± 0.6, *p* = 0.5698, ANOVA). We next used DIV21 neuron–glial cultures to optimize a live-cell imaging approach to measure dendritic spine dynamics. We labeled dendritic spines with an adeno-associated virus (AAV) serotype 1 (AAV1-hSyn-EGFP) and found little to no off-target EGFP expression in glia (Fig. 1*c*). Transduced cultures treated with CXCL12 (20 nM) for 3 h had higher dendritic spine density than the Vehicle group (0.1% BSA/PBS; Fig. 1*d,e*; Vehicle: 6.1 ± 0.3 spines/10 μm vs CXCL12: 7.3 ± 0.3 spines/10 μm, *U* = 483.5, *p* = 0.0053, Mann–Whitney *U* test), demonstrating that CXCL12 can still regulate dendritic spine density in transduced neurons. These initial results laid the foundation to study if CXCL12 regulates spine formation, elimination, and turnover in real time.

We next developed a live imaging timeline based on previous studies of CXCL12 regulation of dendritic spine density (Pitcher et al., 2014; Festa et al., 2020). We imaged transduced neuron–glial cultures over a baseline period of 3 h (Fig. 1*f,j*) and found spontaneous changes in spine density that quickly returned to baseline (Fig. 1*k*). We then treated the groups with either Vehicle or CXCL12 and imaged the cultures every 30 min for 3 h and then every hour for the following 9 h, bringing the total timeline to 12.5 h (Fig. 1*f*). Compared to Vehicle, CXCL12 increased spine density peaked between the 5th and 6th hour—i.e., 3 h after treatment (Fig. 1*k*; 5th hour, Vehicle: −1± 3.1% vs CXCL12: 18.8 ± 4.5%, *p* = 0.0025; 5.5th hour, Vehicle: −0.9 ± 2.9% vs CXCL12: 23.5 ± 3.82%, *p* < 0.0001; 6th hour, Vehicle: 0.09 ± 3.3% vs CXCL12: 20.2 ± 4.8%, *p* = 0.004; *F*_(19, 314)_ = 4.594, interaction *p* < 0.0001, ANOVA). The effect began to wane at 7 h and returned to baseline at the end of the live imaging session at 12.5 h. This led us to speculate whether this effect was due to CXCL12 levels decreasing in culture over time. Therefore, we compared spine density in neuronal cultures treated with a single dose or multiple doses of CXCL12 over 6 h. In alignment with the previous live imaging results, a single CXCL12 treatment significantly increased spine density after 3 h (Fig. 1*g*; Vehicle: 5.6 spines ± 0.25/10 μm vs CXCL12: 6.9 spines ± 0.32/10 μm; *U* = 367.5, *p* = 0.035, Mann–Whitney *U* test), and spine density returned to baseline after 6 h (Fig. 1*h*; Vehicle: 5.3 spines ± 0.3/10 μm vs CXCL12: 5.4 spines ± 0.3/10 μm; *U* = 612.5, *p* = 0.8436, Mann–Whitney *U* test). However, cultures treated with CXCL12 (20 nM) at both 0 and 3 h still showed increased spine density at the 6 h timepoint (Fig. 1*i*; Vehicle: 5.3 spines ± 0.25/10 μm vs CXCL12: 6.5 spines ± 0.28/10 μm; *U* = 435, *p* = 0.0367, Mann–Whitney *U* test), suggesting neurons need a certain threshold of CXCL12 to maintain higher spine density.

We next confirmed that CXCL12 required its cognate receptor CXCR4 to increase dendritic spine density over time. We treated transduced neurons with the specific CXCR4 antagonist AMD3100 (100 ng/ml), both alone and in combination with CXCL12, and quantified spine density at the 5.5th hour during live imaging when CXCL12 effects were most significant. As expected, CXCL12 significantly increased spine density compared with the Vehicle control, and this outcome was blocked by pretreatment with AMD3100 (Fig. 1*l*; Vehicle: 1.8 ± 3.0%; CXCL12: 22.2 ± 4.3%; AMD3100: −6.9 ± 4.0%; CXCL12 + AMD3100: 2.1 ± 4.4%; Vehicle vs CXCL12, *p* = 0.008; CXCL12 vs CXCL12 + AMD3100, *p* = 0.0092; *F*_(3, 20)_ = 9.709, interaction *p* = 0.0004, ANOVA). We also investigated if CXCL12/CXCR4 signaling regulated the rates of spine formation, elimination, and turnover by tracking individual spines over time using MicroDynamix software (MBF Bioscience). First, CXCL12 specifically increased spine formation compared with Vehicle and AMD3100 again blocked this effect (Fig. 1*m*; Vehicle: 1.2-fold ± 0.2; CXCL12: 2.4-fold ± 0.4; AMD3100: 1.4-fold ± 0.2; CXCL12 + AMD3100: 1.2-fold ± 0.3; Vehicle vs CXCL12, *p* = 0.0205; CXCL12 vs CXCL12 + AMD3100, *p* = 0.0244; *F*_(3, 20)_ = 4.643, interaction *p* = 0.0127, ANOVA). At the same time point, neither CXCL12 nor CXCL12 + AMD3100 altered the spine elimination rate compared with Vehicle and other groups (Fig. 1*n*; Vehicle: 1.4-fold ± 0.2; CXCL12: 2.0-fold ± 0.7: AMD3100: 1.4-fold ± 0.4; CXCL12 + AMD3100: 1.9-fold ± 0.6; *F*_(3, 20)_ = 0.3699, interaction *p* = 0.7756, ANOVA). We used spine formation and elimination rates to calculate spine turnover at the same time point and found no significant differences among the various groups. However, there was a trend toward increased turnover in the CXCL12 group (Fig. 1*o*; Vehicle: 1.3-fold ± 0.1; CXCL12: 2.0-fold ± 0.3; Vehicle + AMD3100: 1.4-fold ± 0.3; CXCL12 + AMD3100: 1.6-fold ± 0.2; Vehicle vs CXCL12, *p* = 0.0869; Vehicle vs AMD3100, *p* = 0.98; Vehicle vs CXCL12 + AMD3100, *p* = 0.5564; *F*_(3, 20)_ = 2.045, interaction *p* = 0.1399, ANOVA).

Together, these results show that CXCL12/CXCR4 signaling can increase dendritic spine density via new spine formation, and repeated CXCL12 treatments maintain spine density gains. These results are similar to our in vivo studies, where adult rats given daily intracerebroventricular infusions of CXCL12 (25 ng/dose) showed increased prelimbic cortex spine density over at least 72 h compared with the control (Fig. 1*p*), including increased levels of thin spines (Fig. 1*q*; thin spines; control: 2.1 spines ± 0.18/10 μm; 24 h: 3.47 spines ± 0.17/10 μm, *p* = 0.002; 48 h: 4.09 spines ± 0.24/10 μm, *p* < 0.0001; 72 h: 4.58 spines ± 0.26/10 μm, *p* < 0.0001; *F*_(3, 12)_ = 24.83, interaction *p* < 0.0001, ANOVA) and a corresponding reduction in immature, stubby spines (Fig. 1*r*; stubby spines; control: 1.63 spines ± 0.13/10 μm; 24 h: 1.00 spines ± 0.08/10 μm, *p* = 0.0045; 48 h: 0.61 spines ± 0.11/10 μm, *p* < 0.0001; 72 h: 0.60 spines ± 0.12/10 μm, *p* < 0.0001; *F*_(3, 12)_ = 19.58, interaction *p* < 0.0001, ANOVA). Overall, these results highlight that CXCL12/CXCR4 signaling mediates spinogenesis and dendritic spine plasticity over several hours.

### CXCL12 stabilizes dendritic spines

Newly formed dendritic spines can rapidly engage with a presynaptic area and stabilize over time. As spines stabilize, they grow larger and accumulate synaptic proteins to enhance synaptic transmission. Stable dendritic spines are marked by PSD-95, a primary scaffold protein of the postsynaptic density that supports a variety of synaptic and interspine components. PSD-95 is also associated with spine persistence in vivo (Cane et al., 2014; Taft and Turrigiano, 2014), as a live imaging experiment on mouse layer 2/3 somatosensory cortex neurons found that 20% of newly formed spines have PSDs, and these spines are less likely to be eliminated after 18 h (Cane et al., 2014). Spines with PSD-95 are also more functionally mature and maintain synaptic contacts, which is important for cognitive functions (Taft and Turrigiano, 2014). Therefore, following the discovery of new spine formation, we examined if CXCL12 also stabilized spines by maintaining their expression of PSD-95.

To this end, we first optimized adeno-associated viruses to sequentially label endogenous synaptic PSD-95 (AAV1-Syn-PSD-95.FingR-EGFP, the PSD-95 intrabody) and the overall spine structure (AAV1-hSyn-mScarlet), which allowed us to monitor how PSD-95 levels changed in dendritic spines of dual transduced neurons. Since the PSD-95 intrabody may recognize other MAGUK proteins when overexpressed, including SAP97, SAP102, and PSD-93 (Gross et al., 2013), we first compared the intrabody's EGFP expression to standard immunostaining for PSD-95 in cultured neurons. The intrabody and the standard antibody signal colocalized on almost all PSD-95 puncta within spines, suggesting good specificity for both approaches (Fig. 2*a*; PSD-95 puncta stained by both intrabody and antibody: 89.9 ± 1.21% vs PSD-95 puncta stained only by antibody: 10.1 ± 1.21%; *t*_(4)_ = 80.73, *p* < 0.0001, unpaired *t* test). As an additional control experiment, we examined if the intrabody unduly stabilized PSD-95 on its own, which could bias our studies on spine stabilization. Neurons were transduced at DIV1 with either the intrabody construct or an EGFP control construct (AAV1-hSyn-EGFP) and analyzed for PSD-95 puncta size at DIV21 using the EGFP signal and standard immunostaining for PSD-95. PSD-95 puncta area remained the same in neurons transduced with either construct as well as in untransduced control cultures, suggesting the PSD-95 intrabody does not interfere with endogenous PSD-95 functions (Fig. 2*b,c*; untransduced: 0.16 μm^2^ ± 0.002; EGFP: 0.15 μm^2^ ± 0.004; PSD-95.FingR-EGFP: 0.15 μm^2^ ± 0.002; *H*_(2)_ = 1.935, *p* = 0.3800, Kruskal–Walis test). The PSD-95 intrabody also successfully tracked the trafficking and accumulation of PSD-95 within spines (Fig. 2*d*), providing further confidence that this approach can determine the stability of PSD-95 within spines.

We next analyzed how CXCL12 treatment affected the stability of dendritic spine PSD-95 in dual transduced neurons using a live imaging approach in DIV21 neuron–glial cultures (Fig. 2*e*). Prior to treatments, we imaged neurons over a baseline of 6 h and measured spontaneous changes in PSD-95 puncta area. We then added either CXCL12 (20 nM) or Vehicle (0.1% BSA/PBS) control treatments and imaged the cultures every 3 h for an additional 18 h (Fig. 2*f*). PSD-95 puncta area was similar at 3 h post-treatment (Vehicle, 3rd hour: −0.06-fold ± 0.05 vs CXCL12, 3rd hour: 0.03-fold ± 0.04, *p* = 0.374; *F*_(2, 12)_ = 15.36, time factor *p* = 0.0005, ANOVA). However, CXCL12 stabilized PSD-95 puncta area at later timepoints compared with the Vehicle control group (Fig. 2*g*; Vehicle, 9th hour: −0.12-fold ± 0.04 vs Vehicle, 18th hour: −0.33 fold ± 0.09, *p* = 0.0079; CXCL12, 9th hour: −0.012-fold ± 0.07 vs CXCL12, 18th hour: −0.12 fold ± 0.09, *p* = 0.1729; *F*_(1, 6)_ = 2.810, group factor *p* = 0.1447, ANOVA). CXCL12-treated neurons also had significantly larger PSD-95 puncta areas at the 18th hour post-treatment (Fig. 2*g*; Vehicle, 18th hour: −0.33 fold ± 0.09 vs CXCL12, 18th hour: −0.12 fold ± 0.09, *p* = 0.0362; *F*_(2, 12)_ = 15.36, time factor *p* = 0.0005, ANOVA), further suggesting CXCL12 stabilized PSD-95 within spines for an extended time. As there were no changes in PSD-95 puncta area during the baseline, pretreatment period, it is unlikely that other intrinsic factors contributed to our results (Fig. 2*h*; pre-Vehicle: 0.33 μm ± 0.01 vs pre-CXCL12: 0.32 μm ± 0.01; *U* = 44409, *p* = 0.1558, Mann–Whitney *U* test). Additionally, CXCL12 treatment also led to a slight but significant increase in spine head diameter, an indicator of spine head enlargement and stabilization in DIV21 neuronal cultures (Fig. 2*i*; Vehicle: 0.25 μm ± 0.004 vs CXCL12: 0.27 μm ± 0.004; *D* = 0.05431, *p* = 0.0011, Kolmogorov–Smirnov test). This result was in line with our stabilization experiment results and others showing that spines enlarge their head region prior to recruiting additional synaptic proteins (Bosch et al., 2014) and becoming functional.

We followed up to determine if CXCL12 also increases receptor expression in dendritic spines. DIV21 neuronal cultures were treated with CXCL12 (20 nM) or Vehicle (0.1% BSA/PBS) for 3 h and then fixed for immunostaining. We costained cultures for PSD-95 and the neuronal marker MAP2, followed by a counterstain with fluorophore-conjugated phalloidin to label dendritic spines. We then acquired immunofluorescence images from random areas via confocal microscopy and analyzed the density of spines with PSD-95 punctate staining using Neurolucida 360 software (MBF Bioscience). CXCL12 treatment increased the density of dendritic spines containing PSD-95 within neuronal cultures, further suggesting it helps spines to stabilize (Fig. 3*a*; Overall PSD-95^+^ spines, Vehicle: 2.15 spines ± 0.13/10 μm vs CXCL12: 3.04 spines ± 0.14/10 μm, *p* < 0.0001; *F*_(4, 351)_ = 8.194, interaction *p* < 0.0001, ANOVA). We then reconstructed the overall morphology of these spines and analyzed individual spine types with Neurolucida 360 software. This analysis revealed CXCL12 specifically increased the density of thin spines expressing PSD-95 (Fig. 3*b*; thin, PSD-95^+^ spines, Vehicle: 1.56 spines ± 0.1/10 μm vs CXCL12: 2.2 spines ± 0.1/10 μm, *p* < 0.0001, *F*_(4, 351)_ = 8.194, interaction *p* < 0.0001, ANOVA), but not mushroom spines, stubby spines, or filopodia (Fig. 3*b*). Other studies report that phosphorylation of PSD-95 on serine 295 helps recruit PSD-95 to the PSD region and recruit AMPA receptors to the spine head (Kim et al., 2007; Vallejo et al., 2017) further stabilizing the spine. Notably, CXCL12 treatment also increased the density of spines containing phosphorylated PSD-95^S295^ which was also most prevalent in thin spines (Fig. 3*c,d*; overall PSD-95^S295+^ spines, Vehicle: 3.8 spines ± 0.2/10 μm vs CXCL12: 4.8 spines ± 0.2/10 μm, *p* = 0.0036; thin, PSD-95^S295+^ spines, Vehicle: 2.18 spines ± 0.13/10 μm vs CXCL12: 2.98 spines ± 0.18/10 μm, *p* = 0.0002; *F*_(4, 370)_ = 7.046, interaction *p* < 0.0001, ANOVA). We also found a corresponding increase in the density of spines expressing the AMPA receptor subunit GluA1 in the spine head, again most prevalent in thin spines (Fig. 3*e,f*; overall GluA1^+^ spines, Vehicle: 2.5 spines ± 0.13/10 μm vs CXCL12: 3.7 spines ± 0.18/10 μm, *p* < 0.0001; thin, GluA1^+^ spines, Vehicle: 1.68 spines ± 0.09/10 μm vs CXCL12: 2.47 spines ± 0.16/10 μm, *p* < 0.0001; *F*_(4, 389)_ = 7.104, interaction *p* < 0.0001, ANOVA).

These data suggest that CXCL12 helps stabilize thin dendritic spines by enhancing their levels of synaptic components and stabilizing the synaptic scaffolds that support their function. CXCL12 may thus promote the maturation process of newly formed spines and exert a broader stabilizing effect on the overall spine population.

### CXCL12 increases presynaptic proteins associated with dendritic spines

Mature dendritic spine types are often used as a readout for excitatory synapses, as they house the synapse's postsynaptic component. However, synapses have a corresponding presynaptic compartment as well, which houses different synaptic proteins. Therefore, we checked if CXCL12 could increase clusters of pre- and postsynaptic proteins on dendritic spines. We treated neuronal cultures with CXCL12 (20 nM, 3 h) or Vehicle followed by immunostaining for PSD-95, the presynaptic marker vesicular glutamate transporter 1 (vGluT1), and the neuronal marker MAP2. Cultures were also counterstained with phalloidin to visualize dendritic spines, which allowed us to measure PSD-95 and vGluT1 staining on dendritic spines with ImageJ's cell counter plugin (Fig. 4*a*). As expected from our previous results, CXCL12 increased overall spine density and the density of spines containing PSD-95 as well as spines apposed to vGluT1 puncta. CXCL12 also strongly increased the density of spines with vGluT1/PSD-95 clusters, further suggesting that CXCL12 effects on dendritic spines translate to full synapses (Fig. 4*b*; overall spines, Vehicle: 6.48 spines ± 0.29/10 μm vs CXCL12: 8.79 spines ± 0.32/10 μm, *p* < 0.0001; overall PSD-95^+^ spines, Vehicle: 5.29 spines ± 0.22/10 μm vs CXCL12: 7.51 spines ± 0.31/10 μm, *p* < 0.0001; overall vGluT1^+^-apposed spines, Vehicle: 4.90 spines ± 0.22/10 μm vs CXCL12: 7.2 spines ± 0.30/10 μm, *p* < 0.0001; vGluT1^+^-only apposed spines (PSD95^−^), Vehicle: 0.5 spines ± 0.06/10 μm vs CXCL12: 0.53 spines ± 0.6/10 μm, *p* > 0.9999; overall vGluT1^+^/PSD-95^+^ synapse clusters on spines, Vehicle: 4.37 spines ± 0.19/10 μm vs CXCL12: 6.66 spines ± 0.31/10 μm, *p* < 0.0001; *F*_(4,340)_ = 7.243, interaction *p* < 0.0001, ANOVA). Notably, CXCL12 also increased the percentage of vGluT1/PSD-95 clusters on the overall population of spines (Fig. 4*c*; Vehicle: 68 ± 1.6% vs CXCL12: 74.9 ± 1.5%, *p* = 0.0027, unpaired *t* test) and specifically on more stabilized spines containing PSD-95 (Fig. 4*d*; Vehicle: 82.8 ± 1.2% vs CXCL12: 88 ± 1%, *p* = 0.0013, unpaired *t* test). Together, these results make clear that CXCL12 regulation of dendritic spines not only affects the postsynaptic compartment but also leads to a corresponding increase of presynaptic components interacting with these spines, suggesting these new spines are part of functional synapses.

Given the positive results with excitatory synapses, we also checked if CXCL12 has corresponding effects on inhibitory synapses in culture. This experiment measured clustered staining of inhibitory pre- and postsynaptic markers along dendrites, as inhibitory synapses typically lack dendritic spines. Following treatments with CXCL12 (20 nM, 3 h), the CXCR4 antagonist AMD3100 (100 ng/ml, 3 h), or the combination, we immunostained each culture for MAP2, the presynaptic protein synapsin 1 and inhibitory postsynaptic protein gephyrin, and then analyzed the number of synapsin 1/gephyrin clusters using ImageJ's SynapseJ plugin (Fig. 4*e*). These studies showed no changes in density of inhibitory synapses in all treatment groups (Fig. 4*f*; Vehicle: 2.27 ± 0.22 synapse clusters/10 μm^2^; CXCL12: 2.51 ± 0.23 synapse clusters/10 μm^2^; AMD3100: 2.02 ± 0.17 synapse clusters/10 μm^2^; AMD3100 + CXCL12: 2.57 ± 0.25 synapse clusters/10 μm^2^; *F*_(3,139)_ = 1.306, interaction *p* = 0.2749; ANOVA), suggesting CXCL12's effects on synapse density are specific to excitatory synapses with postsynaptic dendritic spines. These studies were controlled to ensure they analyzed the same overall area of MAP2 staining in each group (Fig. 4*g*), and each group showed similar overall numbers of synapsin 1/gephyrin clusters in the analyzed images (Fig. 4*h*). Overall, these data show CXCL12 selectively increases the density of excitatory pre- and postsynaptic proteins on dendritic spines, further suggesting these spines are part of functional excitatory synapses.

### CXCL12 subtly promotes thin spine clustering

We next examined if CXCL12 regulated a type of spine plasticity called spine clustering, as anatomical clustering of spines on a dendrite can begin to facilitate nonlinear synaptic transmission events that affect local neuronal networks (Kastellakis et al., 2015). Our approach relied on a nearest neighbor index (NNI) computational method that measures the extent of spine clustering while controlling for differences in spine density among groups (Fig. 5*a*). Our enhanced NNI method compared the average distances between all dendritic spines and between different spine types, allowing us to determine how specific spine types clustered after treatments. Interestingly, CXCL12 treatment (20 nM, 3 h) did not alter clustering among the entire population of spines (Fig. 5*b*; NNI cluster threshold = 0; Vehicle: all spines, 0.27 ± 0.02 vs CXCL12: all spines, 0.21 ± 0.02, *p* = 0.0737, *t*_(227)_ = 1.797, unpaired *t* test) but it reduced the average distance between thin spines in DIV21 neuronal cultures (Fig. 5*c*; Vehicle: thin spines, 0.28 ± 0.03 vs CXCL12: thin spines, 0.16 ± 0.03, *p* = 0.0116, *t*_(227)_ = 2.546, unpaired *t* test). These results further suggest that CXCL12 preferentially regulates thin spines and may help them to incorporate into local networks via anatomical spine clustering.

### CXCL12 regulates local network activity in multielectrode array cultures

As CXCL12 regulates elements of spine formation and stabilization, it may also regulate neuronal circuits and contribute to network-level activity. This hypothesis is supported by our previously published data where CXCL12 treatment improved cognitive flexibility in HIV-1 transgenic rats (Festa et al., 2020) and numerous other reports on the neuromodulatory actions of CXCL12/CXCR4 signaling in different brain regions (Guyon, 2014; Pitcher et al., 2014). Since neuronal network changes likely underlie improved cognitive performance, we examined if CXCL12 regulated neuronal network activity parameters over time in cultured cortical neurons. These studies used multielectrode arrays (MEAs) to capture network outputs and investigate neural network dynamics over time and pharmacological treatments.

We first established the developmental trajectory of neuronal activity within cortical neuron–glial MEA cultures and identified optimal time points for pharmacological interventions. We recorded cortical network activity weekly for 4 weeks and quantified overall activity, synchronous network activity, and characteristics of synchronous network bursts such as burst duration and frequency. MEA cultures steadily developed neuronal activity over time, as shown in a representative spike heatmap from one culture (Fig. 6*a*). MEA cultures developed structured network activity after DIV15, with increased overall spiking activity (Fig. 6*b*; DIV8: 3,488 spikes ± 1464/MEA; DIV15: 10,633 spikes ± 1446/MEA; DIV22: 26,001 spikes ± 4,237/MEA; DIV28: 51,375 spikes ± 8018/MEA; for all comparisons *p* < 0.001; *F*_(25,75)_ = 3.138, interaction *p* < 0.0001, ANOVA), increased network bursts (Fig. 6*c*; DIV15: 11 bursts ±2.16/MEA; DIV22: 25.19 bursts ± 2.58/MEA; DIV28: 46.35 bursts ± 4.93/MEA; for all comparisons *p* < 0.0001; *F*_(25,75)_ = 3.34, interaction *p* < 0.0001, ANOVA), increased spike frequency in network bursts (Fig. 6*d*; DIV15: 479.10 Hz ± 78.68/MEA; DIV22: 685.80 Hz ± 43.26/MEA; DIV28, 682.90 Hz ± 40.83/MEA; DIV15 vs DIV22, *p* < 0.0001; DIV22 vs DIV28, *p* = 0.002; *F*_(25,75)_ = 3.27, interaction *p* < 0.0001, ANOVA), and a higher percentage of spikes within network bursts (Fig. 6*e*; DIV15: 32.93% ± 4.92/MEA; DIV22: 73.80% ± 4.10/MEA; DIV28: 86.69% ± 2.36/MEA; for all comparisons *p* < 0.0001; *F*_(25,75)_ = 4.789, interaction *p* < 0.0001, ANOVA), suggesting network development over time. Additionally, network burst duration remained the same after DIV15 (Fig. 6*f*; DIV15: 479.10 ms ± 78.68/MEA; DIV22: 685.80 ms ± 43.26/MEA; DIV28: 682.90 ms ± 40.83/MEA; DIV15 vs DIV22, *p* = 0.1704; DIV22 vs DIV 28, *p* > 0.9999; DIV15 vs DIV28, *p* = 0.1665; *F*_(25,75)_ = 0.8034, interaction *p* = 0.7255, ANOVA). Collectively, these pilot studies indicate that cortical cultures exhibit active structured networks with a robust phenotype by DIV28, allowing us to study how CXCL12 regulates neuronal networks in cortical cultures with established network connectivity.

We next treated DIV28 MEA cultures with either CXCL12 (20 nM) or Vehicle control (0.1% BSA/PBS) to examine CXCL12 effects on network activity. Each MEA culture was recorded at 0, 3, 6, 24, and 48 h after the initial treatment. Interestingly, CXCL12 altered the structure of network bursts by increasing spike frequency within network bursts 3 h post-treatment (Fig. 6*g*; Vehicle: 3rd hour, 0.98-fold ± 0.02, vs CXCL12: 3rd hour, 1.04-fold ± 0.02, *p* = 0.0288; Vehicle: 24th hour, 0.99-fold ± 0.03 vs CXCL12: 24th hour, 1.17-fold ± 0.05, *p* = 0.0017; *F*_(4, 204)_ = 3.297, interaction *p* = 0.0121, ANOVA). This increasing trend reached the highest point in the CXCL12-treated group 24 h post-treatment (Fig. 6*g*; CXCL12, baseline vs 24th hour, *p* = 0.007; *F*_(2.923, 149.0)_ = 3.098, time factor *p* = 0.0298, ANOVA), with no change in synchronous burst duration (Fig. 6*h*; Vehicle vs CXCL12; *F*_(4, 250)_ = 0.08102, *p* = 0.9881, ANOVA). CXCL12 did not affect overall network activity, network burst number, the interval between network bursts, average spikes in a network burst, or the percentage of spikes in network bursts at the designated timepoints (data not shown). These results suggest that CXCL12 effects on spine plasticity translate to distinct network-level activity via increasing neuron firing within synchronous network bursts.

### CXCR4 expression in neuronal subpopulations

We were curious if CXCL12 acted on particular types of neurons to drive downstream effects on dendritic spines and neuronal network activity. Recent single-cell RNA-seq studies report that CXCL12's canonical receptor CXCR4 is enriched in GABAergic inhibitory cortical neurons, specifically those expressing somatostatin (SST), while CXCL12 is mostly enriched in excitatory cortical neurons in adult mice (Tasic et al., 2016; Yao et al., 2021). This opened an intriguing possibility that CXCL12 may engage inhibitory neurons to promote dendritic spine plasticity on local excitatory neurons. We started to investigate this possibility by first detecting which neurons in our primary culture system expressed CXCR4 and comparing our results to previous reports of CXCR4 expression in the cerebral cortex in vivo observed by in situ hybridization (Stumm et al., 2007) and single-cell RNA sequencing (Tasic et al., 2016).

We first validated the monoclonal CXCR4 (UMB2) antibody via Western blot. This antibody recognizes the unphosphorylated C-terminus of mouse and human CXCR4 (Mueller et al., 2013; Abe et al., 2014), so we validated this antibody for the rat isoform of CXCR4. We expressed rat CXCR4-EGFP (rCXCR4-EGFP) or C-terminal truncated CXCR4-EGFP (rCXCR4-ΔCT-EGFP) in HEK293T cells and found the antibody recognized full-length but not the C-terminal truncated rCXCR4 construct that lacks the antibody epitope (Fig. 7*a*). rCXCR4-EGFP and rCXCR4-ΔCT-EGFP were recognized by an EGFP antibody at their expected molecular weights, confirming that both proteins were expressed at suitable levels for detection. We next tested the CXCR4 (UMB2) antibody for immunostaining using primary rat neuronal cultures nucleofected with an mDlx enhancer-driven CXCR4 construct that specifically overexpressed CXCR4 in inhibitory neurons. As expected, the CXCR4 antibody–stained pattern closely matched that of glutamate decarboxylase 67 (GAD67)-positive inhibitory cortical neurons (Fig. 7*b*).

Using the validated CXCR4 antibody, we next tested whether the cell types expressing CXCR4 in cortical cultures match those identified in the cortex (Stumm et al., 2007). Although some reports show CXCR4 expression in pure astrocyte cultures (Bezzi et al., 2001), others did not find Cxcr4 transcripts in astrocytes under basal and postischemic conditions (Stumm et al., 2002). Therefore, we first determined cellular CXCR4 expression in neuron–glial cultures compared with neuronal cultures. Our neuron–glial cultures at DIV21 did not show CXCR4 immunoreactivity in GFAP-positive astrocytes (Fig. 7*c*), and both culture types showed a similar overall number of CXCR4-positive neurons via MAP2 staining (Fig. 7*d*; neuron–glial culture: 4.4% ± 1.1 vs neuronal culture: 6.1% ± 1.1, *p* = 0.3017; *t*_(31)_ = 1.05, unpaired *t* test), suggesting that glia do not alter neuronal CXCR4 expression in our cortical cultures. We then investigated whether CXCR4 was expressed in inhibitory and/or excitatory neurons. These experiments identified the type of neuron by expression of GAD67, an important GABA-producing enzyme highly expressed in GABAergic inhibitory neurons with little to no expression in excitatory neurons (Becchetti et al., 2012; Sahara et al., 2012). In line with previously reported single-cell RNA-seq data (Tasic et al., 2016), GAD67-positive inhibitory neurons were significantly more likely to express CXCR4 than GAD67-negative excitatory neurons in both culture conditions (Fig. 7*e,g*; percentage of CXCR4^+^ neurons in cultures; neuron–glial cultures: inhibitory neurons: 11.2% ± 1.85 vs excitatory neurons: 2% ± 0.4, *p* = 0.0058; neuronal cultures: inhibitory neurons: 13.0% ± 3.5 vs excitatory neurons: 2.4% ± 0.9, *p* = 0.0016; *F*_(1, 32)_ = 0.1089, interaction *p* = 0.7436, ANOVA). Although SST-positive neurons were slightly more prevalent in pure neuronal cultures (Fig. 7*i*; percentage of SST^+^ neurons in cultures: neuron–glial: 4.78% ± 0.46; neuronal: 7.11% ± 0.90, *p* = 0.03; *t*_(16)_ = 2.3, unpaired *t* test), these neurons were the most likely to express CXCR4 in both culture systems and no group differences were detected (Fig. 7*f,h*; CXCR4^+^; SST^+^ neuron percentage in culture; neuron–glial: 35.4% ± 5.8; neuronal: 43.8% ± 10.5, *p* = 0.4964; *t*_(16)_ = 0.69, unpaired *t* test). These findings show that CXCR4 is expressed in a major subset of inhibitory neurons in our culture systems that could regulate many excitatory neurons via their extending axons.

To explore this possibility, we knocked down Cxcr4 in inhibitory neurons using an AAV construct expressing EGFP with the mDlx enhancer, which targets inhibitory neurons (Dimidschstein et al., 2016). The mDlx construct expression was highly restricted to neurons stained with the inhibitory marker GAD67 in neuron–glial cultures, as expected (Fig. 8*a*; EGFP^+^ and GAD67^+^ cells, 90% of total counted cells, 3,376 cells counted, *N* = 3). We modified the construct by inserting a miR30a cassette designed to knock down Cxcr4 expression in inhibitory neurons (AAV-mDlx-EGFP-mir30a-shCxcr4) along with control constructs (AAV-mDlx-EGFP-mir30a-scramble and AAV-mDlx-EGFP). Using CXCR4 protein immunostaining (Fig. 8*b*) in neuron–glial cultures, we found that shCxcr4 specifically reduced CXCR4 protein expression within individual GFP-expressing somata (GAD67-positive somata used for untransduced controls; Fig. 8*c*; untransduced: 18,565 arbitrary unit(AU)/µm^2^ ± 905; EGFP: 16,791 AU/µm^2^ ± 978; scramble: 15,576 AU/µm^2^ ± 957; shCxcr4: 10,978 AU/µm^2^ ± 698; untransduced vs EGFP, *p* = 0.5394; EGFP vs scramble, *p* = 0.7636; scramble vs shCxcr4, *p* = 0.0013; EGFP vs shCxcr4, *p* < 0.0001; *F*_(3, 123)_ = 12.93, interaction *p* < 0.0001, ANOVA), and shCxcr4 was effective regardless of baseline CXCR4 levels in individual somata (Fig. 8*d*). On average, the shCxcr4 group had ∼50% less CXCR4 protein in inhibitory neurons compared with untransduced cultures, while EGFP and scramble transduction groups were similar to untransduced neurons (Fig. 8*e*; EGFP: 0.92 fold ± 0.06; scramble: 0.88 fold ± 0.04; shCxcr4: 0.58 fold ± 0.04; EGFP vs scramble, *p* = 0.8729; EGFP vs shcxcr4, *p* = 0.0101; scramble vs shCxcr4, *p* = 0.0174; *F*_(2, 6)_ = 11.97, interaction *p* = 0.0080, ANOVA). We next investigated if CXCL12 could still regulate spine density in the CXCR4-deficient cortical cultures. Cultures were sequentially transduced with controls or the Cxcr4 knockdown construct on DIV1, followed by the transduction of AAV-hSyn-mScarlet to label spines on DIV14. CXCL12 increased spine density as expected in DIV21 neuron–glial cultures transduced with either control construct, but interestingly, CXCL12 failed to increase overall spine density in cultures with an inhibitory neuron-specific CXCR4 knockdown (Fig. 8*f*; untransduced, Vehicle: 5.67 spines ± 0.19 μm vs CXCL12: 7.87 spines ± 0.24/10 μm, *p* < 0.0001; EGFP, Vehicle: 6.47 spines ± 0.23/10 μm vs CXCL12: 9.06 spines ± 0.29/10 μm, *p* < 0.0001; scramble, Vehicle: 6.48 spines ± 0.31/10 μm vs CXCL12: 8.02 spines ± 0.29/10 μm, *p* < 0.0001; shCxcr4, Vehicle: 6.89 spines ± 0.31/10 μm vs CXCL12: 6.17 spines ± 0.25/10 μm, *p* = 0.0588; intragroup comparisons of CXCL12-treated, untransduced vs shCxcr4, *p* < 0.0001; EGFP vs shCxcr4, *p* < 0.0001; scramble vs shCxcr4, *p* < 0.0001; *F*_(3, 278)_ = 15.22, interaction *p* < 0.0001, ANOVA). These results are also consistent with CXCL12's preferential regulation of thin spines, as CXCL12 increased thin spine density in all groups except the shCxcr4 inhibitory neuron-specific knockdown group (Fig. 8*g*; untransduced, Vehicle: 3.57 spines ± 0.15/10 μm vs CXCL12: 5.04 spines ± 0.15/10 μm, *p* < 0.0001; EGFP, Vehicle: 4.17 spines ± 0.20/10 μm vs CXCL12: 5.96 spines ± 0.25/10 μm, *p* < 0.0001; scramble, Vehicle: 4.13 spines ± 0.22/10 μm vs CXCL12: 5.27 spines ± 0.19/10 μm, *p* < 0.0001; shCxcr4, Vehicle: 4.33 spines ± 0.25/10 μm vs CXCL12: 4.04 spines ± 0.18/10 μm, *p* = 0.3091; intragroup comparisons of CXCL12-treated, untransduced vs shCxcr4, *p* = 0.0031; EGFP vs shCxcr4, *p* < 0.0001; scramble vs shCxcr4, *p* = 0.001; *F*_(3, 278)_ = 10.35, interaction *p* < 0.0001, ANOVA). These results suggest the CXCL12/CXCR4 pathway must be activated in GABAergic inhibitory neurons to regulate dendritic spines on nearby excitatory neurons. Overall, our results show that CXCL12 regulates elements of spine plasticity associated with learning and memory, and these effects on spines translate to structured network activity that could improve cognition and cognitive processes.

## Discussion

Our work shows that the chemokine CXCL12 is not limited to simply increasing dendritic spine density in cortical neurons—it also facilitates a host of other dynamic processes within dendritic spines allowing them to grow, integrate into neuronal networks, and modulate network-level activity. CXCL12 achieves these outcomes by modulating thin spines, a dynamic spine type that may underlie learning and memory via synaptic plasticity (Bourne and Harris, 2007). Cortical neurons treated with CXCL12 gradually formed new spines over several hours, which was sustained with repeated treatments. CXCL12 also increased the density of thin spines with synaptic markers, including PSD-95 and the AMPA receptor subunit GluR1, better preserved PSD95 expression within spines, and increased pre- and postsynaptic protein interactions on spines. Thin spines were also slightly closer together on the dendrite, suggesting they may be amenable to clustered plasticity processes and network-level changes that drive learning. Indeed, CXCL12 increased neurons’ firing frequency within synchronous network bursts, in line with the higher density of functional spine types in these cultures. Intriguingly, CXCR4 was only expressed in ∼5% of neurons in our cultures, suggesting a small cadre of neurons drives widespread spine plasticity. CXCR4 was particularly enriched in inhibitory neurons, which lack dendritic spines but can directly innervate the dendrites of nearby excitatory neurons (Chiu et al., 2013; Higley, 2014; Song et al., 2021). These neurons were key intermediates of CXCL12 signaling, as a CXCR4 knockdown construct targeting inhibitory neurons blocked CXCL12 from increasing dendritic spine density. Overall, the results demonstrate that CXCL12/CXCR4 chemokine signaling promotes spine plasticity processes associated with learning and memory (Festa et al., 2020), and the underlying mechanism involves network-level changes driven by specific kinds of neurons.

This study follows up rigorous work showing that CXCL12/CXCR4 signaling increases cortical dendritic spine density across distinct experimental systems, from pure primary neuronal cultures to intact rodent models (Pitcher et al., 2014; Festa et al., 2020). Moreover, CXCL12 reverses spine deficits in the prelimbic cortex of HIV-1 transgenic rats and improves their prelimbic cortex–mediated task performance, further suggesting the chemokine restores network-level processes relevant to learning (Festa et al., 2020). This is in line with studies that report CXCL12/CXCR4 signaling improves cognitive performance in other models of brain disease (Capsoni et al., 2017; Trousse et al., 2019), suggesting our results may also inform CNS therapeutic strategies more broadly. CXCL12's efficacy is particularly striking considering only a small percentage of neurons in our culture system express its receptor CXCR4. However, this pattern of CXCR4 expression is in line with other in situ hybridization studies in developing rat cortical neurons (Stumm et al., 2003, 2007) and single-cell RNA-seq results from adult mouse cortical neurons (Tasic et al., 2016; Yao et al., 2021). RNA-seq data also show that mice restrict CXCR4 expression to specific kinds of cortical neurons, notably including a subset of somatostatin interneurons (Tasic et al., 2016). Of note, somatostatin interneurons are highly prevalent in the human prefrontal cortex (Anderson et al., 2020; Banovac et al., 2022), placing these cells in a region known to regulate learning and memory processes. CXCL12 reportedly modulates neurotransmission and neuronal function in other brain areas as well (Chalasani et al., 2003; Guyon et al., 2006; Bhattacharyya et al., 2008; Khan et al., 2008), further supporting that CXCL12/CXCR4 signaling is an important neuromodulatory pathway that persists into adulthood and is conserved across species. Our future work aims to validate these findings in live human brain tissues (Van Duyne et al., 2024) to determine if CXCL12 effects on spine dynamics and network function translate to the more complex human brain.

Intriguingly, the small group of CXCR4-expressing cortical neurons in our cultures included excitatory and inhibitory neurons, suggesting that CXCL12 signaling may activate different pathways that coalesce to regulate dendritic spine dynamics. For example, CXCR4 signaling on excitatory neurons may directly regulate their actin cytoskeleton, which is a critical component of dendritic spine structure and dynamic functions (Hotulainen and Hoogenraad, 2010). We reported that CXCR4 quickly activates a Rac1/PAK1/cofilin pathway in primary cortical neurons, and downstream Rac1 signaling was necessary to restore spine density in the prelimbic cortex of HIV-1 transgenic rats and improve their cognitive outcomes (Festa et al., 2020). Similarly, CXCL12 can also activate Rho/mDia signaling in cerebellar granule neurons, and subsequent actin polymerization allows their axons to lengthen via chemotactic axon guidance (Arakawa et al., 2003). These short-term effects might be a more subtle extension of CXCL12's well-known chemotactic properties (Sánchez-Alcañiz et al., 2011), where instead of cell bodies navigating a chemokine gradient, cellular processes navigate the extracellular space to establish new connections. In contrast, CXCR4 signaling on inhibitory neurons must use a different approach to regulate dendritic spines, as most of these neurons lack dendritic spines or have very few compared with pyramidal neurons (Kawaguchi et al., 2006). Our data suggest that CXCL12 controls inhibitory neuron activity over a longer term to create network-level conditions that stabilize spines on excitatory neurons. This is supported by our multielectrode array studies where CXCL12 gradually increased spikes in structured network bursts at early timepoints, but the largest effects occurred 24 h after treatment. Notably, this time point is after CXCL12-mediated spine formation returned to baseline levels, suggesting the restructured network connections were functionally mature and active. These effects may be driven by CXCR4-expressing somatostatin interneurons, as they directly innervate excitatory neuron dendrites and precisely tune inhibition in dendritic domains (Riedemann, 2019). Other work suggests CXCR4 signaling interacts with GABAergic systems and neurons in various brain regions (Guyon et al., 2006; Heinisch and Kirby, 2010; Ardelt et al., 2013; Guyon, 2014), but it remains unclear how CXCL12 regulation of dendritic spines controls overall network dynamics in the prefrontal cortex. We are currently investigating these areas using in vivo and ex vivo models.

CXCL12 effects on dendritic spine dynamics are intriguingly similar to other high-profile compounds that restore cognitive function. For example, ketamine and psilocybin rapidly increase dendritic spine formation in neurons of the medial frontal cortex and prelimbic area (Phoumthipphavong et al., 2016; Moda-Sava et al., 2019; Shao et al., 2021), which relieves depressive symptoms and stress-related deficits. Notably, the restored spines were long-lasting and required to sustain ketamine's long-term antidepressant effects (Moda-Sava et al., 2019). Additionally, CXCL12 and other psychoplastogens can increase spine density independent of task-related learning, a significant factor that drives activity-dependent spine plasticity. Both treatments seem to modify basal spine plasticity thresholds and initiate plasticity events rather than merely support or reinforce existing events. These results describe multiple ways to harness spine plasticity processes to effectively treat several neurocognitive disorders, and CXCL12/CXCR4 signaling represents an endogenous mechanism that could be tapped to restore these fundamental processes. Leveraging this pathway might also avoid some of the well-known side effects of psychoplastogens (Olson, 2018; Vargas et al., 2021), which could reduce abuse potential and accelerate approval of new CNS therapeutics.

Evidence suggests neurodegenerative disorders may also be amenable to therapeutic strategies that reverse dendritic spine deficits. Several groups report ways to recover lost spines in models of Alzheimer's disease (Smith et al., 2009) and traumatic brain injury (Xiong et al., 2019), highlighting the continued importance of dendritic spine health across CNS diseases (Smith et al., 2009). These include BDNF/TrkB agonists and insulin receptor agonists (Nagahara et al., 2009; Massa et al., 2010; Ettcheto et al., 2021), which both activate Rac1 and lead to actin polymerization and increased spine density (Ma et al., 2008; Yan et al., 2016). Thus, Rac1-mediated actin remodeling seems to be an important process for spine dynamics that is also activated by CXCL12/CXCR4 signaling (Festa et al., 2020). Other treatments that stabilize actin could make spines more resilient, as the pan-ROCK inhibitor, fasudil, prevented spine loss from amyloid-beta toxicity (Henderson et al., 2019). Though this approach may interfere with new spine formation, it further highlights how actin-modifying pathways can restore cognition or slow disease onset. Interestingly, Alzheimer's disease may eliminate spines in clusters (Mijalkov et al., 2021), so CXCL12's modest effect on thin spine clusters could be therapeutically relevant. However, much work remains to fully understand if CXCL12 can improve spine dynamics and cognitive functions in disorders other than neuroHIV.

CXCL12/CXCR4 signaling likely engages multiple pathways that converge to help form new spines, remove fruitless spines, stabilize existing spines, and potentially cluster synaptic activity, all of which shape how neuronal networks function and adapt to complex stimuli. Healthy spine plasticity processes underlie our ability to learn and remember information and successfully navigate everyday life (Heck and Santos, 2023), so it is critically important to understand how these processes work and how they go awry in neurocognitive disorders. Continued work in this domain holds promise to discover cross-disease therapeutic strategies for a variety of neurocognitive disorders that lack effective treatments, including adjuvant strategies to help repair network connectivity and function. The CXCL12/CXCR4 chemokine axis can help identify these kinds of therapies and further our understanding of the endogenous mechanisms that neurons use to control their elegantly dynamic connections.
